# Colonization and metabolite profiles of homologous, heterologous and experimentally evolved algal symbionts in the sea anemone *Exaiptasia diaphana*

**DOI:** 10.1038/s43705-022-00114-7

**Published:** 2022-03-30

**Authors:** Sarah Jane Tsang Min Ching, Wing Yan Chan, Alexis Perez-Gonzalez, Katie E. Hillyer, Patrick Buerger, Madeleine J. H. van Oppen

**Affiliations:** 1 School of BioSciences, University of Melbourne, Parkville, VIC Australia; 2 Melbourne Cytometry Platform, University of Melbourne, Parkville, VIC Australia; 3 Department of Microbiology and Immunology, The University of Melbourne, at The Peter Doherty Institute of Infection and Immunity, Parkville, VIC Australia; 4 CSIRO Land and Water, Dutton Park, Brisbane, QLD Australia; 5 Applied Biosciences, Macquarie University, North Ryde, NSW Australia; 6 Australian Institute of Marine Science, Townsville, QLD Australia

**Keywords:** Symbiosis, Microbiome, Metabolomics

## Abstract

The sea anemone, *Exaiptasia diaphana*, is a model of coral-dinoflagellate (Symbiodiniaceae) symbiosis. However, little is known of its potential to form symbiosis with *Cladocopium*—a key Indo-Pacific algal symbiont of scleractinian corals, nor the host nutritional consequences of such an association. Aposymbiotic anemones were inoculated with homologous algal symbionts, *Breviolum minutum*, and seven heterologous strains of *Cladocopium* C1^acro^ (wild-type and heat-evolved) under ambient conditions. Despite lower initial algal cell density, *Cladocopium* C1^acro^-anemeones achieved similar cell densities as *B. minutum*-anemones by week 77. Wild-type and heat-evolved *Cladocopium* C1^acro^ showed similar colonization patterns. Targeted LC-MS-based metabolomics revealed that almost all significantly different metabolites in the host and Symbiodiniaceae fractions were due to differences between *Cladocopium* C1^acro^ and *B. minutum*, with little difference between heat-evolved and wild-type *Cladocopium* C1^acro^ at week 9. The algal fraction of *Cladocopium* C1^acro^-anemones was enriched in metabolites related to nitrogen storage, while the host fraction of *B. minutum*-anemones was enriched in sugar-related metabolites. Compared to *B. minutum*, *Cladocopium* C1^acro^ is likely slightly less nutritionally beneficial to the host under ambient conditions, but more capable of maintaining its own growth when host nitrogen supply is limited. Our findings demonstrate the value of *E. diaphana* to study experimentally evolved *Cladocopium*.

## Introduction

Like corals, the sea anemone *Exaiptasia diaphana* is a cnidarian that establishes symbiosis with dinoflagellates in the family Symbiodiniaceae. In this symbiosis, the algae translocate products of carbon fixation and nitrogen assimilation as glucose, glycerol, amino acids, alanine and/or organic acids to the host [1–3], and gain host-derived inorganic nutrients such as carbon dioxide, ammonium, amino acids, lipids and fatty acids [4]. Symbiodiniaceae loss in corals due to ocean warming (i.e., coral bleaching) has significant negative impacts on coral health and the persistence of coral reef ecosystems [5, 6], highlighting the importance of studying the coral-Symbiodiniaceae symbiosis. Compared to corals, however, *E. diaphana* is fast growing, easy to maintain and to render aposymbiotic (i.e., free of algal symbionts), and it can survive longer when bleached. These characters allow researchers to easily establish an *E. diaphana* population, manipulate their Symbiodiniaceae community and to examine cellular processes that would otherwise be challenging to study with corals [7]. Hence, *E. diaphana* is a common a model for coral-Symbiodiniaceae symbiosis.

*E. diaphana* has been used to examine the flexibility of algal symbiont-host pairings [8, 9], and the physiological [10, 11], transcriptomic [12], metabolic [12, 13] and proteomic [14] consequences of associating with homologous and/or heterologous (i.e., non-native) algal symbionts under ambient and/or elevated temperatures. Eleven Symbiodiniaceae genera (i.e., *Breviolum, Cladocopium, Durusdinium, Effrenium, Fugacium, Freudenthalidium, Gerakladium, Halluxium, Miliolidium, Philozoon, Symbiodinium*) have been formally described [15–18]. For Indo-Pacific *E. diaphana*, their homologous (i.e., native) Symbiodiniaceae are predominately *Breviolum minutum* (ITS2: B1) [19], although *Cladocopium* and *Durusdinium* are occasionally found in low abundance [20]. Atlantic *E. diaphana* natively associate with *Symbiodinium linucheae* (ITS2: A4) and *B. minutum* and occasionally with *Cladocopium* [19].

*Cladocopium* spp. are the most widely distributed algal symbionts of Indo-Pacific scleractinian corals [21] and are found in >150 cnidarian species on the Great Barrier Reef (GBR) [22]. Although some heterologous algal symbionts can colonize aposymbiotic *E. diaphana* [8, 9, 11, 12, 14, 23, 24], colonization success of *Cladocopium* spp. is generally poor [11, 25] and these are therefore often excluded from *E. diaphana* experiments [8, 12]. However, most studies only monitored host algal cell density up to a few weeks post inoculation [23]. Nevertheless, a few studies have demonstrated short-term [9] and long-term (>1 year) [23] successful colonization of aposymbiotic *E. diaphana* by *Cladocopium* (ITS2: C1).

All *E. diaphana* studies so far focus on wild-type Symbiodiniaceae (i.e., Symbiodiniaceae that have been growing under long-term ambient conditions), and none have explored the potential of *E. diaphana* as a model to study experimentally evolved Symbiodiniaceae. Experimental evolution has been shown to enhance growth, photo-physiological performance and/or the upper thermal limit in many marine microalgal species [26]. For example, experimentally evolved *Cladocopium* C1^acro^ maintained positive growth and produced less reactive oxygen species (ROS) than wild-type *Cladocopium* C1^acro^ under elevated temperatures (31 °C) [27, 28]. These experimentally evolved heat-tolerant symbionts (hereafter refer to as “heat-evolved” symbionts) can potentially be used to inoculate corals to improve their thermal tolerance [29]. Buerger et al. [28] demonstrated that heat-evolved *Cladocopium* C1^acro^ can colonize aposymbiotic coral larvae, where three of the ten strains tested conferred enhanced thermal bleaching tolerance on the larvae compared to the wild-type *Cladocopium* C1^acro^ while seven did not. However, coral larvae and adults differ significantly in physiology, hence more studies are required to examine the potential benefits and drawbacks of associating with heat-evolved Symbiodiniaceae, particularly in the adult host stage. The values of *E. diaphana* as a coral model would be enhanced significantly if it can form symbiosis with heat-evolved Symbiodiniaceae and this sea anemone serves as a model for these investigations.

Bi-directional exchange of organic and inorganic compounds is critical to the success and stability of the cnidarian-dinoflagellate symbiosis [1]. Different Symbiodiniaceae strains may translocate different types (e.g., glycerol vs. lipids) and quantities of photosynthate to the host [1], affecting the host’s nutritional potential and overall fitness. While *E. diaphana* can be rendered aposymbiotic and be colonized by heterologous Symbiodiniaceae, heterologous associations can be less beneficial to the host than homologous symbioses [8, 10, 12]. However, the nutritional consequences of *E. diaphana* associated with heterologous *Cladocopium*—a key algal symbionts in Indo-Pacific corals, and heat-evolved *Cladocopium*—a potential candidate to improve cnidarian thermal tolerance, remain unknown.

This study aims to examine (1) whether *E. diaphana* from the GBR, which harbours *B. minutum* as its homologous symbiont, can form a stable symbiosis with heterologous wild-type and heat-evolved *Cladocopium* C1^acro^, and (2) the nutritional consequences of the respective *Cladocopium* C1^acro^ associations with specific focus on central carbon metabolism. Since glucose is a key form in which Symbiodiniaceae translocate fixed carbon to the cnidarian host [2, 30], particular focus is given to sugar-related metabolites.

## Materials and methods

### Experimental organisms

GBR-sourced *E. diaphana* (Genotype AIMS4) were obtained from the National Sea Simulator at the Australian Institute of Marine Science (AIMS) in Townsville (Queensland). Anemones were maintained under 27 °C at 30 µmol photons m^−2^ s^−1^ (12:12 h, light: dark) [7] and their homologous algal symbiont is *B. minutum* [7]. They were fed *ad libitum* with freshly hatched *Artemia* nauplii 5 days a week prior to the experiment to establish sufficient biomass, and were fed twice a week during the experiment. Aposymbiotic anemones were produced using a modified menthol-bleaching method [31] (Supplementary Methods 1.1). Eight different monoclonal Symbiodiniaceae strains were used to inoculate the aposymbiotic anemones (Table 1, hereafter referred to as Symbiodiniaceae treatments): the homologous *B. minutum* (hereafter referred to as B1), wild-type *Cladocopium* C1^acro^ (WT10; formerly known as *Cladocopium goreaui* [32]), and six heat-evolved *Cladocopium* C1^acro^ strains (SS) which were derived from the same monoclonal mother culture as WT10, three of which conferred enhanced bleaching tolerance on coral larvae (SS1, SS7 and SS8, jointly referred to as SS+) and three which did not (SS3, SS5 and SS9, jointly referred to as SS−) [28]. Cultures were grown under their respective temperatures (Table 1) at 30–60 µmol photons m^−2^ s^−1^ (12:12 h, light: dark) in 1% IMK culture medium, and were obtained from long-term laboratory cultures established at AIMS in 2011.

**Table 1:** Details of Symbiodiniaceae strains used in this study.

Species	Strain	Group	Details	Culture ID	ITS2	°C^a^	Original host	Host origin
*B. minutum*	B1	B1	Homologous	SCF 127-01	B1	27	*E. diaphana*	Central GBR, Australia
*Cladocopium* C1^acro^	WT10	WT10	Heterologous, wild-type	SCF 055-01.10	C1	27	*Acropora tenuis*	Magnetic Island, Australia
*Cladocopium* C1^acro^	SS1	SS+	Heterologous, heat-evolved, conferring^b^	SCF 055-01.01	C1	31	*Acropora tenuis*	Magnetic Island, Australia
*Cladocopium* C1^acro^	SS7	SS+	Heterologous, heat-evolved, conferring^b^	SCF 055-01.07	C1	31	*Acropora tenuis*	Magnetic Island, Australia
*Cladocopium* C1^acro^	SS8	SS+	Heterologous, heat-evolved, conferring^b^	SCF 055-01.08	C1	31	*Acropora tenuis*	Magnetic Island, Australia
*Cladocopium* C1^acro^	SS3	SS-	Heterologous, Heat evolved, non-conferring^b^	SCF 055-01.03	C1	31	*Acropora tenuis*	Magnetic Island, Australia
*Cladocopium* C1^acro^	SS5	SS−	Heterologous, heat-evolved, non-conferring^b^	SCF 055-01.05	C1	31	*Acropora tenuis*	Magnetic Island, Australia
*Cladocopium* C1^acro^	SS9	SS−	Heterologous, heat-evolved, non-conferring^b^	SCF 055-01.09	C1	31	*Acropora tenuis*	Magnetic Island, Australia

^a^The temperature at which the cultures were maintained at.

^b^“Heat-evolved” refers to a strain that has been experimentally evolved under elevated temperatures as per Chakravarti et al. [27]; “conferring” refers to a strain that conferred enhanced bleaching tolerance on coral larvae and “non-conferring” refers to a strain that did not confer enhanced bleaching tolerance on coral larvae as per Buerger et al. [28].

### Inoculation of aposymbiotic anemones

A total of 480 similar-sized aposymbiotic anemones (3–4 mm oral disk diameter, *n* = 60 anemones per Symbiodiniaceae treatment) were starved for nine days before Symbiodiniaceae inoculation. One anemone was placed in each well of 12-well plates, and each was inoculated by pipetting 1 mL of 1 × 10^6^ algal cells onto its oral disk, followed by 40 µL of freshly hatched *Artemia* nauplii to encourage phagocytosis. The anemones were exposed to the algal inocula for 24 h before seawater change. A second inoculation was performed 48 h later following the same procedure. The number of weeks mentioned throughout this study refers to the weeks since first inoculation. Seventy-two anemones were not inoculated and kept as aposymbiotic controls. After inoculation, anemones were fed *Artemia* twice a week as per previous studies [8, 9, 12, 13, 24].

### Symbiodiniaceae cell density and identity

Four anemones per Symbiodiniaceae treatment were sampled at week 2, 3, 4, 5, 6, 9, 21, 24 and 77 for Symbiodiniaceae cell counts using cytometery (Supplementary Methods 1.2). To confirm that the Symbiodiniaceae in anemones post inoculation matched the inoculum and that no cross contamination has occurred, three sets of samples were collected for ITS2 metabarcoding: (1) 1 mL of each of the eight algal inocula (*n* = 2 per Symbiodiniaceae treatment), (2) aposymbiotic anemones collected at week 21 (*n* = 6), and (3) 100 µL of Symbiodiniaceae collected from anemones at week 9 (*n* = 4 per Symbiodiniaceae treatment), all of which were snap frozen and stored in −80 °C until DNA extraction, PCR amplification and library preparation (Supplementary Methods 1.3). Illumina MiSeq v3 sequencing was conducted at the Walter and Eliza Hall Institute and raw sequences were submitted to SymPortal [33] for Symbiodiniaceae community analysis.

### Anemone survival, dry weight and metabolite sample processing

Twelve anemones of each of the eight Symbiodiniaceae treatments were assessed for survival at week 1, 2, 3, 4, 5, and 9. At week 9, 12 anemones per Symbiodiniaceae treatment were sampled for targeted metabolomics analysis (12 anemones × 8 treatments = 96 total). To avoid contamination from *Artemia* proteins and metabolites, anemones were starved for a week before sampling, when they were snap frozen in liquid nitrogen and stored at −80 °C. Two anemones of the same Symbiodiniaceae treatment were combined into a single sample to provide sufficient biomass for the analysis. Samples were homogenized and centrifuged to separate the host and Symbiodiniaceae fractions (Supplementary Methods 1.4). Each fraction was freeze dried to obtain its dry weight, then extracted and analyzed on the Agilent 1290 Infinity II UHPLC with an Agilent 6470 Triple Quadrupole LC-MS system (Supplementary Methods 1.5). The analysis followed the Agilent metabolomics dynamic MRM (dMRM) method where a curated database with retention times and optimized MS/MS acquisition parameters of ~200 central carbon metabolites are specified. Samples were run in two batches (batch one: B1, WT10, SS− (SS5), SS+ (SS8); batch two: SS− (SS3, SS9) and SS+ (SS1, SS7)).

### Statistical analysis—Symbiodiniaceae cell density and host dry weight

Symbiodiniaceae cell density and dry weight data were analyzed in R (version 3.6.1) [34] using the car package [35] and visualized with the ggplot2 package [36]. Each dataset was tested for normality with the Shapiro test [37] and homogeneity of variance with Levene’s test [38] and log transformed if necessary to meet the assumptions of an analysis of variance (ANOVA). One-way ANOVA was used to test the difference in Symbiodiniaceae cell density and host dry weight between Symbiodiniaceae treatment groups (B1, WT10, SS+, SS−) and Tukey’s pairwise comparisons were then conducted.

### Statistical analysis—metabolomics

The raw data were: (1) blank corrected, (2) normalized to internal standards and (3) normalized to the host dry weight (for the host fraction), or Symbiodiniaceae dry weight (for the Symbiodiniaceae fraction). The data were then batched-corrected (automated, least distance amongst batches) and zero-treated (missing value = 1/5 of the Limit of Detection) in MetaboAnalyst 5.0 [39]. See Supplementary Methods 1.6, Figs. S1 and S2 for batch correction details. Data analysis was performed in MetaboAnalyst 5.0 separately for the host and Symbiodiniaceae fractions. Log transformation and pareto scaling were applied to each dataset, and data normality and homogeneity were visually confirmed. The analyses were repeated without scaling and the outcomes remained the same. Note that fold changes (FC) reported throughout this study were calculated in MetaboAnalyst 5.0 using the data with no transformation or scaling.

PCAs and PLS-DAs were generated using all the 207 central carbon metabolites. Metabolites that were significantly different between Symbiodiniaceae treatments were identified with a one-way ANOVA and visualized with a heatmap using Euclidean distance and Ward clustering algorithm. Pairwise comparisons were then examined for the host and Symbiodiniaceae fractions (i.e., *B. minutum* vs *Cladocopium* C1^acro^, *Cladocopium* C1^acro^ WT10 vs SS, and *Cladocopium* C1^acro^ SS− vs SS+), and *t*-tested. *P* values were corrected using the Benjamini–Hochberg method [40] and a metabolite was considered significant when the *p*_adj_ < 0.05 and FC > 1.3. To identify the pathways that the significant metabolites were linked to, MetaboAnalyst’s Pathway Analysis function [41] was used with *Plasmodium falciparum* 3D7 library (the option most closely related to Symbiodiniaceae [42]). For both the host and Symbiodiniaceae fractions, only the *B. minutum* vs *Cladocopium* C1^acro^ comparison yielded a sufficient number of metabolites for meaningful pathway analysis (see Results), hence the analysis was only conducted for this pair.

A metabolite was classified as sugar-related if it was a disaccharide (i.e., two molecules of glucose or one molecule of glucose with, e.g., a galactose), monosaccharide (i.e., single molecular sugar), amino sugar, sugar acid, sugar alcohol or intermediate sugar metabolite (Supplementary Methods 1.7). Anemones with more Symbiodiniaceae biomass may receive more sugar-related metabolites translocated by the symbionts. While *B. minutum*-anemones had higher algal cell density than *Cladocopium* C1^acro^-anemones at the time of sampling for metabolomics (see Results), the *B. minutum* cells are smaller than *Cladocopium* C1^acro^ cells [2, pers. observ.], therefore Symbiodiniaceae dry weight was used as a proxy of Symbiodiniaceae biomass. Two different normalization methods were applied for all sugar-related metabolites in the host fraction: (1) normalized to the host dry weight only, and (2) normalized to the host and Symbiodiniaceae dry weight.

## Results

### Symbiodiniaceae cell density and identity

**Fig. 1:**
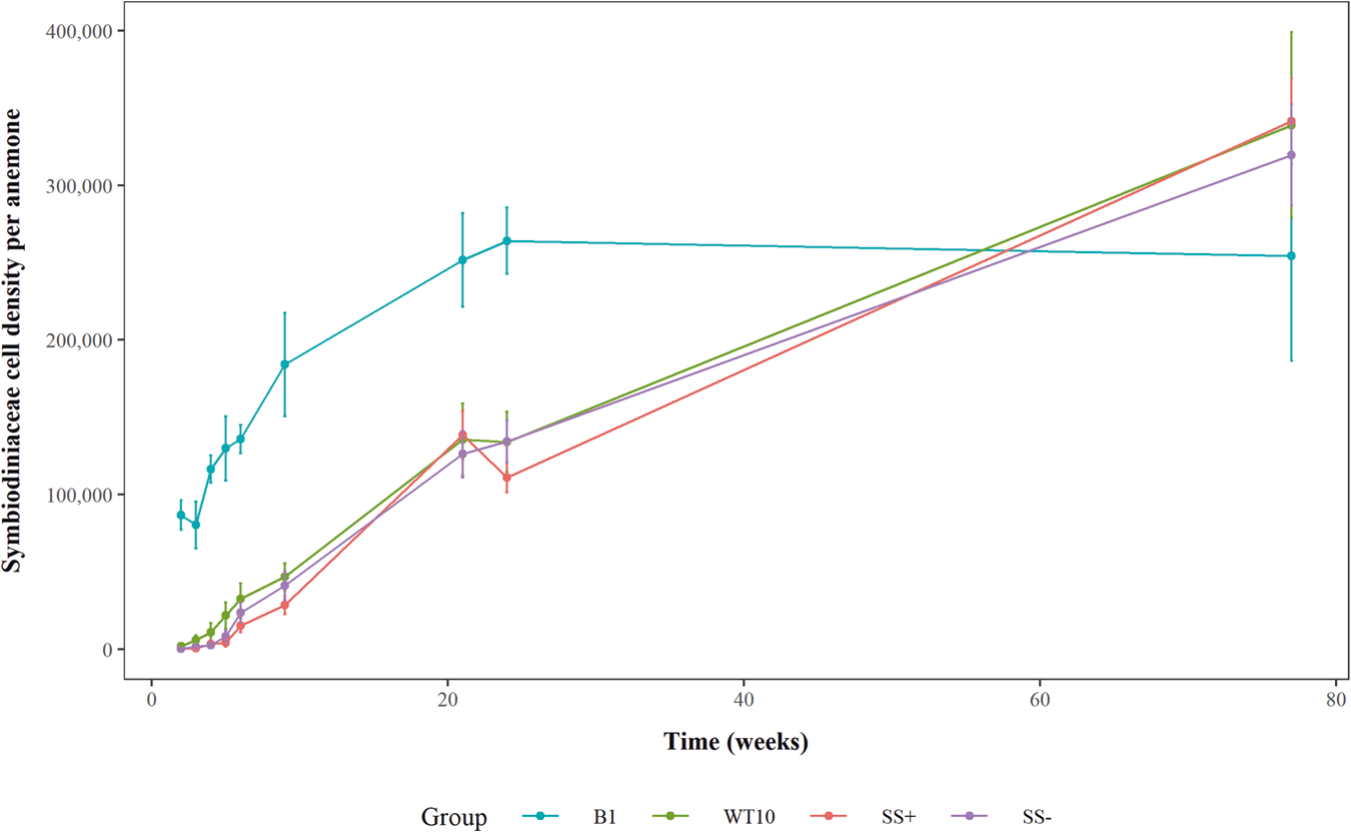
Symbiodiniaceae cell density per anemone over the 77 weeks following initial inoculation. Error bars represent one standard error. B1 = *B. minutum*, WT10 = wild-type *Cladocopium* C1^acro^, SS− = non-conferring heat-evolved *Cladocopium* C1^acro^, SS+ = conferring heat-evolved *Cladocopium* C1^acro^.

At week 9 (when the metabolic samples were taken), anemones of different Symbiodiniaceae treatments differed significantly in Symbiodiniaceae cell density (*F*_3,28_ = 7.36, *p* < 0.001). Pairwise comparisons indicated that *B. minutum*-anemones had higher cell densities than *Cladocopium* C1^acro^ SS+ and SS− anemones, while cell densities of all *Cladocopium* C1^acro^-anemones (WT10, SS+, SS−) were the same (Fig. 1, S3, Table 2, S1). By week 77, however, anemone groups no longer differed in Symbiodiniaceae cell density (*F*_3,28_ = 0.66, *p* = 0.587) (Fig. 1, Table 2). ITS2 metabarcoding data showed the identity of the Symbiodiniaceae in the anemones matched that of the inocula (Fig. S4, Supplementary Results 1.1).

**Table 2:** Symbiodiniaceae cell density per anemone at week 9 and 77 following initial inoculation.

Group	Strain	Culture ID	Week 9- mean	Week 9- 1SE	Week 77- mean	Week 77- 1SE	*n*
B1	B1	SCF 127-01	1.84 × 10^5^	33,528	2.54 × 10^5^	67,646	4
WT10	WT10	SCF 055-01.10	4.67 × 10^4^	8855	3.39 × 10^5^	59,993	4
SS+	SS1	SCF 055-01.01	2.24 × 10^4^	6697	4.00 × 10^5^	44,022	4
	SS7	SCF 055-01.07	3.68 × 10^4^	7818	3.49 × 10^5^	40,967	4
	SS8	SCF 055-01.08	2.65 × 10^4^	15,020	2.75 × 10^5^	48,945	4
SS−	SS3	SCF 055-01.03	3.90 × 10^4^	9007	2.96 × 10^5^	42,670	4
	SS5	SCF 055-01.05	3.57 × 10^4^	7683	3.77 × 10^5^	76,123	4
	SS9	SCF 055-01.09	4.89 × 10^4^	28,198	2.85 × 10^5^	49,608	4

B1 = *B. minutum*, WT10 = wild-type *Cladocopium* C1^acro^, SS− = non-conferring heat-evolved *Cladocopium* C1^acro^, SS+ = conferring heat-evolved *Cladocopium* C1^acro^.

### Anemone survival and dry weight

At week 9, anemones of different Symbiodiniaceae treatments had 100% survival (Table S2). Dry weight of the anemone host at week 9 was significantly different between Symbiodiniaceae treatment groups (*F*_3,44_ = 7.14, *p* < 0.001). Pairwise comparisons indicated the host dry weight of the *Cladocopium* C1^acro^ groups (WT10, SS + , SS−) did not differ from each other, but they were on average ~34% lower than that of the *B. minutum*-anemones (Fig. S5, Table S3). While dry weight of aposymbiotic anemones was not measured, we observed that *Cladocopium* C1^acro^-anenomes and *B. minutum*-anemones were both noticeably larger in size than aposymbiotic anemones.

### Metabolomics—anemone host fraction

**Fig. 2:**
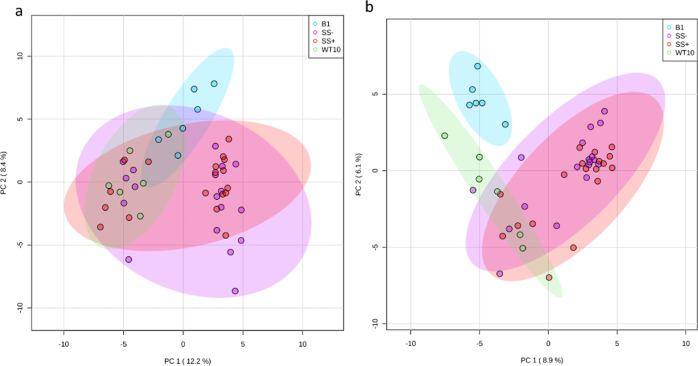
PCAs using all 207 central carbon metabolites. **a** PCA of the host fraction (normalized to host dry weight) measured at week 9. **b** PCA of the Symbiodiniaceae fraction (normalized to Symbiodiniaceae dry weight) measured at week 9. B1 *B. minutum*, WT10 wild-type *Cladocopium* C1^acro^, SS− non-conferring heat-evolved *Cladocopium* C1^acro^, SS+ conferring heat-evolved *Cladocopium* C1^acro^.

The three *Cladocopium* C1^acro^ groups (i.e., anemones colonized by heterologous Symbiodiniaceae wild-type WT10, heat evolved SS+ and SS−) overlapped almost completely in the PCA generated from the host fraction for all 207 central carbon metabolites, and most *B. minutum*-anemone (i.e., anemones colonized by homologous Symbiodiniaceae) replicates did not separate from *Cladocopium* C1^acro^-anemones either (Fig. 2a). The PLS-DA did not pass quality control (permutation *p* = 0.09, Q2 < 0.2; indicating low predictive ability) and is hence not used for interpretation (Fig. S6). ANOVA identified 23 metabolites (~11%) that varied significantly in relative abundance among groups (B1, WT10, SS−, SS+) and *t*-tests showed this pattern was driven by differences between the *B. minutum* and *Cladocopium* C1^acro^ groups (WT10, SS−, SS+) (Table 3, S4, Fig. S7a). Out of these 23 metabolites, 19 and four were enriched in *B. minutum*- and *Cladocopium* C1^acro^-anemones, respectively, with the metabolites d-maltose and cellobiose the most significantly enriched in *B. minutum*-anemones (Table 3, Fig. S7a). None of the metabolites showed a significant difference between wild-type *Cladocopium* C1^acro^ (WT10) and heat-evolved *Cladocopium* C1^acro^ (SS), nor between the heat-evolved SS− and SS+ (Table S4). The heatmap based on the 23 significant host fraction metabolites showed a similar pattern to the PCA, although with a clearer separation between *B. minutum* and all *Cladocopium* C1^acro^ groups (Fig. S7a). The 23 significant metabolites are linked to seven pathways, but only one metabolite match was found in each pathway (Table S5). Similar t-test results were found when only batch one data from the host fraction was used (Table S6).

**Table 3:** Metabolites with significantly different relative abundances in the host fraction.

Normalization^a^	Metabolite	t.stat	*p*_adj_	FC^b^	log2(FC)
Host dry weight	**d-maltose**	49.3	<0.001	7.45	2.90
	**Cellobiose**	47.7	<0.001	7.46	2.90
	**Melibiose**	6.0	<0.001	8.61	3.11
	Nicotinic acid	5.8	<0.001	8.48	3.08
	O-phosphorylethanolamine	4.4	0.002	5.95	2.57
	2-isopropylmalic acid	4.0	0.007	4.73	2.24
	Cis-aconitic acid	−4.0	0.007	0.09	−3.43
	l-kynurenine	−3.7	0.013	0.10	−3.37
	**d-glucose 6-phosphate**	3.6	0.015	1.95	0.96
	**l-sorbose**	3.6	0.015	4.26	2.09
	Pyridoxal hydrochloride	3.6	0.015	2.81	1.49
	4-pyridoxic acid	3.5	0.015	2.42	1.28
	Myo-inositol	3.6	0.015	4.03	2.01
	Salicylic acid	3.5	0.015	2.34	1.23
	Uridine	3.5	0.015	1.88	0.91
	Isopentyl acetate	3.3	0.022	8.89	3.15
	3-dehydroshikimic acid	−3.3	0.024	0.60	−0.75
	Guanine	−3.2	0.032	0.48	−1.04
	Thymidine	3.1	0.033	1.96	0.97
	Citric acid / dl-isocitric acid	3.1	0.035	8.55	3.10
	**l-arabinose**	3.1	0.036	5.02	2.33
	Dl-2-aminoadipic acid	3.0	0.045	2.76	1.47
	l-hydroxyglutaric acid	2.9	0.049	1.93	0.95
Host and Symbiodiniaceae dry weight	**d-maltose**	6.9	<0.001	3.27	1.7
	**Cellobiose**	6.9	<0.001	3.27	1.7
	**Melibiose**	4.0	0.003	3.72	1.9
	**d-mannose**	−2.8	0.035	0.18	−2.5

*T*-test results and fold changes of the significant metabolites between the *B. minutum* and *Cladocopium* C1^acro^-anemones (WT10, SS−, SS+) at week 9 from the host fraction (normalized to the host dry weight or normalized to the host and Symbiodiniaceae dry weight^a^). Metabolites in bold face are sugar-related.

^a^Two different normalization methods were used: (1) normalized to the host dry weight only, and (2) normalized to the host and Symbiodiniaceae dry weight. Note that the double normalization was only applied to sugar-related metabolites under the assumption that these were translocated to the host by Symbiodiniaceae.

^b^A fold change (FC) > 1 suggests that this metabolite was enriched in *B. minutum*-anemones, whereas a FC < 1 indicates that this metabolite was enriched in the *Cladocopium* C1^acro^-anemones. For example, a FC value of 0.1 means that this metabolite was 10 times more abundant in the *Cladocopium* C1^acro^-anemones than the *B. minutum*-anemones.

The 207 central carbon metabolites in this study comprise 20 sugar-related metabolites and one sugar-related pathway (Supplementary Methods 1.7). There was no difference between wild-type and heat-evolved *Cladocopium* C1^acro^-anemones, nor between the homologous *B. minutum*- and *Cladocopium* C1^acro^-anemones in this pathway (Table S5). When these 20 sugar-related metabolites in the host fraction were normalized to both host and Symbiodiniaceae dry weight, six out of the 20 sugar-related metabolites were enriched in *B. minutum*-anemones compared to *Cladocopium* C1^acro^-anemones (by ~2 to 9-fold, Table 3). However, when these 20 sugar-related metabolites were normalized to both host and Symbiodiniaceae dry weight, only three out of the 20 sugar-related metabolites were enriched in *B. minutum*-anemones (by ~3 to 4-fold), and one sugar-related metabolite (D-Mannose) was enriched in *Cladocopium* C1^acro^-anemones (by ~5-fold) (Table 3).

### Metabolomics—Symbiodiniaceae fraction

In contrast to the host fraction, PCA of the Symbiodiniaceae fraction showed complete separation between the *B. minutum* and *Cladocopium* C1^acro^ groups (WT10, SS+, SS−) (Fig. 2b). Heat-evolved *Cladocopium* C1^acro^ (SS− and SS+) overlapped with each other, and a few of the WT10 replicates clustered with the heat-evolved SS− and SS+ (Fig. 2b, S7b). The PLS-DA did not pass quality control (permutation *p* = 0.45, Q2 ~ 0.3; indicating low predictive ability) and is therefore not discussed further (Fig. S8). A total of 24 metabolites showed significant differences in relative abundance among groups (B1, WT10, SS−, SS+) based on ANOVA (Fig. S7b). When WT10, SS− and SS+ were combined as *Cladocopium* C1^acro^ and compared with *B. minutum*, *t*-tests suggested 27 metabolites were significantly different (Table 4). In contrast to the host fraction for which metabolites were mostly enriched in *B. minutum*-anemones, in the Symbiodiniaceae fraction most of the significantly different metabolites (17 out of 19) were enriched in *Cladocopium* C1^acro^ (Table 4). Uric acid was the metabolite with the greatest difference and was >10 times more abundant in *Cladocopium* C1^acro^ algal fraction than in *B. minutum* (Table 4). These 27 metabolites were linked to 13 pathways, three of which had two metabolite matches (i.e., purine metabolism, glyoxylate and dicarboxylate metabolism, and pyrimidine metabolism) (Table S7), and all were enriched in *Cladocopium* C1^acro^ groups compared with *B. minutum* (Fig. S9). Three amino acids (n-formyl-l-tyrosine, o-phospho-l-serine, and l-arginine) were significantly enriched in heat-evolved *Cladocopium* C1^acro^ compared to wild-type, two of which (o-phospho-l-serine, and l-arginine) had a high FC of >300 (Table S8). T-tests results of the Symbiodiniaceae fraction using batch one data only were similar to those based on both batches, although a smaller number of significant metabolites was found (Table S9).

**Table 4:** Significantly different metabolites in the Symbiodiniaceae fraction.

Metabolite	t.stat	p_adj_	FC^a^	log2(FC)
Uric acid	−8.26	<0.001	0.09	−3.40
Trehalose 6-phosphate	−5.11	0.001	0.13	−2.97
Creatinine	−4.46	0.004	0.27	−1.91
l-citrulline	−4.14	0.008	0.15	−2.75
Quinic acid	−4.03	0.009	0.21	−2.24
Adipic acid	−3.36	0.020	0.40	−1.33
Citramalic acid	−3.51	0.020	0.23	−2.15
Cytidine	−3.61	0.020	0.19	−2.40
d-pantothenic acid	−3.55	0.020	0.26	−1.96
l-arabitol / xylitol	−3.34	0.020	0.17	−2.51
l-cystine	−3.47	0.020	0.23	−2.11
Lipoamide	−3.35	0.020	0.05	−4.44
Melibiose	3.36	0.020	4.20	2.07
O-phosphorylethanolamine	3.45	0.020	8.01	3.00
Taurine	−3.40	0.020	0.47	−1.10
Trans-trans muconic acid	−3.55	0.020	0.22	−2.19
Uridine triphosphate (UTP)	−3.62	0.020	0.02	−5.49
Cis-aconitic acid	−3.22	0.025	0.07	−3.90
2–3-dihydroxyisovalerate	−3.12	0.029	0.01	−6.77
3-hydroxyphenylacetic acid	−3.13	0.029	0.02	−5.38
Guanine	−3.14	0.029	0.28	−1.83
O-succinyl-l-homoserine	−3.07	0.032	0.15	−2.71
Cytidine triphosphate (CTP)	−3.01	0.037	0.07	−3.86
Isopentenyl pyrophosphate	−2.96	0.040	0.46	−1.11
2–4-quinolinediol	−2.91	0.041	0.14	−2.82
l-glutamic acid	−2.92	0.041	0.49	−1.02
Xanthine	−2.93	0.041	0.47	−1.10

*T*-test results and fold changes of the 27 significant metabolites between the *B. minutum* and *Cladocopium* C1^acro^ (WT10, SS−, SS+) groups at week 9 from the Symbiodiniaceae fraction (normalized to Symbiodiniaceae dry weight).

^a^A fold change (FC) > 1 suggests that this metabolite was enriched in *B. minutum*-anemones, whereas a FC < 1 indicates that this metabolite was enriched in the *Cladocopium* C1^acro^-anemones. For example, a FC value of 0.1 means that this metabolite was 10 times more abundant in the *Cladocopium* C1^acro^-anemones than the *B. minutum*-anemones.

## Discussion

### Heterologous wild-type and heat-evolved *Cladocopium* C1^acro^ can establish a functional symbiosis with *E. diaphana*

Despite being heterologous algal symbionts, our results show that wild-type and heat-evolved *Cladocopium* C1^acro^ can establish a functional symbiosis with *E. diaphana*. While Symbiodiniaceae cell density in all *Cladocopium* C1^acro^*-*anemones was lower than in *B. minutum*-anemones initially, all anemones achieved the same cell density by week 77. With a few exceptions [12], the homologous *B. minutum* is generally more successful in colonizing aposymbiotic anemones than heterologous Symbiodiniaceae [8, 9, 11, 12, 14, 24, 43, 44]. For example, *B. minutum* had faster colonization and higher cell densities in Indo-Pacific anemones than the heterologous *S. microadriaticum* (ITS2: A1), *Durusdinium trenchii* (ITS2: D1a), *Effrenium voratum* (ITS2: E1) and *Cladocopium* spp. (ITS2: C3) [11]. Medrano et al. [24] reported a fourfold higher cell density in anemones colonized by *B. minutum* compared to *D. trenchii*, although Matthews et al. [12] found similar cell density between the two by week 5 post inoculation.

Successful long-term symbiosis between *E. diaphana* and the heterologous *Cladocopium* is rare. Of the few known cases of *Cladocopium* uptake, Chen et al. [23] reported successful colonization of bleached anemones with *C. goreaui* for >1 year, and the *Cladocopium* cells were transmitted to their asexual offspring produced via pedal laceration. Another *Cladocopium* species (ITS2: C3) was observed to successfully colonize anemones initially, but their cell density declined rapidly to ~2.0 × 10^4^ cells mg^−1^ protein by week 8 post inoculation [11]. *Cladocopium* was only able to colonize the oral disk and tentacles of the anemones initially (as opposed to the entire body as with the homologous *B. minutum*), and was restricted to the oral disk only by week 8 [11]. Successful short-term (30 days) colonization of *Cladocopium* C1^acro^ in GBR *E. diaphana* has previously been demonstrated, although the cell density was fluctuating over time [9].

There are several possible explanations for the colonization success of *E. diaphana* by the heterologous wild-type and heat-evolved *Cladocopium* C1^acro^ in this study. Firstly, the heterologous Symbiodiniaceae inocula used in previous studies generally originated from different parts of the world compared to the host [8, 11, 14, 25], except in Tortorelli et al. [9], while both the host and Symbiodiniaceae originated from the central GBR in our study. Allopatric divergence in traits required for symbiosis maintenance (e.g., cell–cell recognition) may have hindered the heterologous algal colonization success, although successful colonization by a heterologous Symbiodiniaceae inoculum originated from regions different to that of the host has been reported in some cases [12, 23]. The field of Symbiodiniaceae-host recognition is still in its infancy, but the role of glycan–lectin interactions [45, 46] and the potential involvement of d-galactose, l-fucose, d-xylose and d-galacturonic acid in the establishment of this mutualism have been shown [45]. Future studies comparing the recognition molecules of Symbiodiniaceae and host from different regions will shed light on the topic of allopatric divergence and Symbiodiniaceae-host compatibility.

Secondly, colonization success of *Cladocopium* C1^acro^ in *E. diaphana* may not have been fully reflected in previous studies due to the relatively short observation periods. At week 9, Symbiodiniaceae cell density observed in our study was consistent with most published works (i.e., lower in the heterologous algae). However, the Symbiodiniaceae cell densities in *B. minutum*-anemones plateaued by week 21–24, whereas they continued to increase in *Cladocopium* C1^acro^-anemones and had achieved the same level as *B. minutum* by week 77. The symbiosis established by wild-type and heat-evolved *Cladocopium* C1^acro^ was functional and healthy, as indicated by the 100% host survival. Conversely, associations with unsuitable heterologous Symbiodiniaceae can result in significant host mortality [9]. Our findings highlight the potential of *E. diaphana* as a model to study cnidarian-dinoflagellate symbiosis with *Cladocopium* C1^acro^, a common and widespread species in Indo-Pacific reef-building corals, as well as with experimentally evolved *Cladocopium* C1^acro^ that may enhance holobiont thermal bleaching tolerance [28].

### Wild-type and heat-evolved *Cladocopium* C1^acro^ had similar colonization rates and sugar translocation ability

Multiple studies have demonstrated that experimental evolution can enhance certain traits of marine microalgae, although trade-offs are sometimes observed [26]. Earlier studies [27, 28] showed that the heat-evolved *Cladocopium* C1^acro^ used in this study were able to maintain positive growth and secreted less ROS under elevated temperature (31 °C) in vitro than their wild-type counterparts. At the same time, heat-evolved *Cladocopium* C1^acro^ grew slower than the wild-type in vitro under ambient conditions [28]. We observed no evidence of trade-offs with *in vitro* growth and ROS secretion in terms of the symbionts’ ability to colonize and translocate sugar to the host, based on the 207 central carbon metabolites examined under ambient condition. It is not known whether the three amino acids enriched in heat-evolved *Cladocopium* C1^acro^ in symbiosis with *E. diaphana* may be related to their enhanced thermal tolerance *in vitro* [28]. Arginine, as an proteinogenic amino acid with the highest nitrogen to carbon ratio, is an ideal organic nitrogen storage in plants [47]. Heat-evolved *Cladocopium* C1^acro^ may have greater ability for organic nitrogen storage, which may enable them to maintain growth under elevated temperatures [48]. However, whether this is beneficial to their host is debatable [49, 50] and requires further testing.

### Heterologous *Cladocopium* C1^acro^ may be slightly less nutritionally beneficial to the *E. diaphana* host than homologous *B. minutum* under ambient conditions

Anemones associated with heterologous algal symbionts have been shown to have lower photosynthesis rates, less growth, pedal laceration [11], carbon translocation [10], and an upregulation of innate immunity responses and lipid catabolism [12]. ^13^C labelling showed that all sugar compounds investigated (i.e., maltose, fructose, sucrose and xylose) were solely present in anemones with the homologous *B. minutum* [51]. None of these sugar compounds were detected in *D. trenchii*-anemones, and these also had a lower amount and diversity of ^13^C-labelled carbohydrates and lipogenesis precursors. The authors therefore concluded that homologous algal symbionts provide greater fitness benefits to the host than heterologous algal symbionts via increased *de novo* glucose synthesis and translocation. Such strong contrast was not observed in our data.

When the relative abundance of sugar-related metabolites was normalized to the host dry weight alone, a few of the sugar-related metabolites were enriched in *B. minutum-*anemones compared to *Cladocopium* C1^acro^-anemones at week 9. This may be a consequence of the higher Symbiodiniaceae densities in *B. minutum-*anemones at the week 9 time point, which may also be responsible for the higher host dry weight at that time point. When the relative abundance of these sugar-related metabolites was normalized to both the host and Symbiodiniaceae dry weight, interestingly, fewer metabolites were enriched in *B. minutum-*anemones compared to *Cladocopium* C1^acro^-anemones, and one sugar-related metabolite was even enriched in *Cladocopium* C1^acro^-anemones. This indicates that sugar translocation per algal biomass of *Cladocopium* C1^acro^ was likely not much lower than that of *B. minutum*. Heterologous *Cladocopium* C1^acro^, therefore, seems slightly less nutritionally beneficial to the host than homologous *B. minutum* under ambient conditions, but likely more nutritionally beneficial to the host than heterologous *D. trenchii*. Several studies have shown that coral dominated by *Durusdinium* grow more slowly [52, 53] and have lower stored lipids [54] and δ13C values [55] than conspecifics dominated by *Cladocopium* in the field; and that *Cladocopium* translocated more carbon to its host compared to *Durusdinium* under ambient conditions [56]. Nevertheless, a few studies observed no difference in the host and symbiont metabolite pool [57], as well as in heterotrophic nutrition [55] between *Durusdinium* and *Cladocopium* dominated corals.

### Heterologous *Cladocopium* C1^acro^ and homologous *B. minutum* differ in nitrogen storage ability

**Fig. 3:**
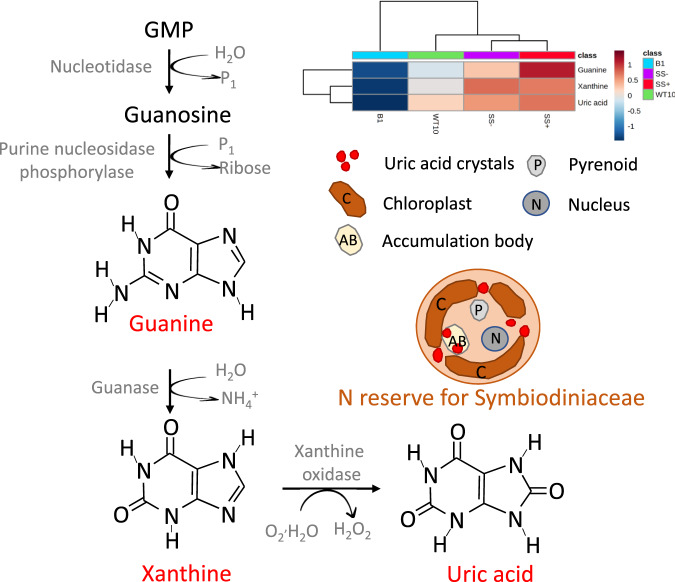
Diagram of a simplified purine degradation pathway with relevance to Symbiodiniaceae nitrogen storage. The red font represents metabolites enriched in the *Cladocopium* C1^acro^ groups (WT10, SS+, SS−) compared to *B. minutum* (B1) and their relative abundances are shown on the heatmap. The locations of uric acid crystals within a Symbiodiniaceae cell as observed in Clode et al. [61] are indicated. The colour scale indicates log2 fold change relative to the mean.

In the Symbiodiniaceae fraction, the two significantly enriched metabolites in the purine metabolism pathways (i.e., guanine and xanthine) and the most significantly enriched metabolite (i.e., uric acid) in the heterologous *Cladocopium* C1^acro^ compared to the homologous *B. minutum* are all linked to nitrogen storage ability. Purine metabolism plays an important role as an ongoing source of nitrogen in plant growth. In the purine degradation pathway, guanase converts guanine to xanthine, and xanthine oxidase catalyzes the oxidation of xanthine to form uric acid as the end product (Fig. 3) [58, 59]. Six genes encoding xanthine oxidase/dehydrogenase have been found in a Symbiodiniaceae (*Fugacium kawagutii*) genome, supporting the ability of these algae to form uric acid [60]. Uric acid functions as a nitrogen reserve in plants and uric acid crystals have been found in Symbiodiniaceae [61] (Fig. 3). When *Exaiptasia* spp. were treated with an inhibitor of xanthine oxidase, the uric acid crystals of their Symbiodiniaceae disappeared within seven days, hence these nitrogen reserves can be mobilized rapidly when required [61].

Nitrogen is vital to the synthesis of amino acids, proteins, nucleotides, nucleic acids, chlorophyll, Rubisco and other enzymes that are involved in carbohydrate production - all of which are critical for cell division and growth [62]. Compared to free-living Symbiodiniaceae, Symbiodiniaceae *in hospite* have higher carbon-to-nitrogen ratios and upregulate multiple transcripts involved in the purine degradation pathway [43]. This suggests Symbiodiniaceae *in hospite* are nitrogen limited [43]; and a coral host can control the growth and therefore population density of its Symbiodiniaceae by limiting nitrogen supply [63, 64]. Under elevated temperatures, Symbiodiniaceae *in hospite* may grow more initially due to higher carbon fixation [50], yet they are unable to use that carbon for their own growth if they are nitrogen limited. When the coral *Plesiastrea versipora* was starved (i.e., no feeding), nitrogen deficiency became apparent in ≥ 4 weeks in their associated Symbiodiniaceae and their photosynthetic rates declined drastically by ~80% [65]. The enhanced nitrogen storage ability of the *Cladocopium* C1^acro^ compared to *B. minutum* suggests that they are likely more capable of maintaining photosynthesis for their own growth.

One *E. diaphana* study showed that heterologous *Durusdinium* cells were more ^15^N-enriched than homologous *Breviolum* cells, yet they provided less carbon to the host under ambient conditions [44]. The extra nitrogen availability to *Durusdinium* may have been a consequence of lower carbon translocation of the algae, that resulted in lesser host usage of its nitrogen re-assimilated from catabolism [44]. The implications of Symbiodiniaceae nitrogen storage ability on the maintenance of their mutualist relations with the host remains unclear and is an important field for future research.

### Conclusions and directions for future research

Several implications relevant to reef restoration and future research have emerged from this study. Firstly, better colonization success and nutrient provisioning may occur when the host and algal symbiont are from the same geographic region. Hence, reef restoration practices that utilize algal symbionts should consider the importance of co-evolution and source algal symbionts from locations near the targeted hosts when possible. Secondly, lower initial host colonization is expected when heterologous algal symbionts are used for reef restoration practices. However, their colonization success will likely improve over time and longer-term monitoring (i.e., >1 year) is important. While the *Cladocopium* C1^acro^ used is this study is a heterologous symbiont of *E. diaphana*, it is the most common Symbiodiniaceae genus in Indo-Pacific scleractinian corals [21] and a homologous symbiont for a wide variety of coral species [22]. Therefore, heat-evolved *Cladocopium* C1^acro^ would likely colonize many coral species at a much faster rate than *E. diaphana*, and potentially conferring bleaching tolerance to the coral hosts.

Evidence so far from the literature and this study suggests that homologous Symbiodiniaceae symbionts are likely more nutritionally beneficial to the host than heterologous symbionts under ambient conditions, yet this dynamic may change under ocean warming which should be a focus of future research. Under elevated temperatures, thermally tolerant heterologous symbionts (e.g., the heat-evolved *Cladocopium* C1^acro^) may become advantageous and more nutritionally beneficial to the host [66, 67]. Due to the significant biomass requirement, omics studies (e.g., metabolomics, proteomics, transcriptomics) on *E. diaphana* are generally limited to ambient temperature only [12, 14, 24], or limited to one *E. diaphana*-Symbiodiniaceae combination when multiple temperatures are applied [13]. In addition to omics studies, future studies on heat-evolved Symbiodiniaceae can also apply tacking methods such as ^13^C labelling, which would provide direct evidence on photosynthate translocation from the algal symbionts to the host. Future studies investigating the physiology and metabolite profiles of anemones associated with homologous and different thermally tolerant heterologous algal symbionts under elevated temperatures will provide invaluable insights for reef restoration initiatives.

## Supplementary information

Supplementary Information

Supplementary data S1

Supplementary data S2

Supplementary data S3

Supplementary data S4

## Data Availability

Metabolic data that have been (1) blank corrected, (2) normalized to internal standards, (3) normalized to the host dry weight (for the host fraction), or Symbiodiniaceae dry weight (for the Symbiodiniaceae fraction), (4) batched-corrected and (5) zero-treated are supplied (host data: Supplementary data S1, Symbiodiniaceae data: Supplementary data S2). Anemone host and Symbiodiniaceae dry weight data (Supplementary data S3), as well as cell density data (Supplementary data S4) are also supplied. Raw sequences of the ITS2 Symbiodiniaceae data are available in GenBank (SAMN22563740- SAMN22563800; project accession no.: PRJNA774436).
