# Supplementary material for: Colonization and metabolite profiles of homologous, heterologous and experimentally evolved algal symbionts in the sea anemone Exaiptasia diaphana
Source: ISME Commun. 2022 Mar 30;2:30. doi: 10.1038/s43705-022-00114-7 (PMC9723793; doi:10.1038/s43705-022-00114-7)
Supplement: Supplementary file 1 — Supplementary Information [file 43705_2022_114_MOESM1_ESM.docx]

**Supplementary information for-**

**Colonization and metabolite profiles of homologous, heterologous and experimentally evolved algal symbionts in the sea anemone *Exaiptasia diaphana***

Sarah Jane Tsang Min Ching*, Wing Yan Chan*, Alexis Perez-Gonzalez, Katie E. Hillyer, Patrick Buerger, Madeleine J. H. van Oppen

*these authors contributed equally to this work

**Supplementary Methods**

**1. 1 Menthol bleaching methods**

To produce aposymbiotic anemones, symbiotic anemones were incubated in seawater with 0.27 mM menthol and 5 µM DCMU for 8 h, followed by seawater with 5 µM DCMU only for 16 h. This 24 h treatment cycle was repeated on four consecutive days a week for five weeks. Seawater used in this study was made with Red Sea SaltTM and reverse osmosis water and maintained at 34 ppt salinity. On day one of the four-day schedule, the anemones were transferred to a 4 ℃ cold room for 2 h. Anemones were not fed during the four-day bleaching in the week, and were fed freshly hatched *Artemia* nauplii only on day five (i.e., the first day without menthol treatment in the week). After five weeks of menthol/DCMU treatment, ~220 anemones were haphazardly selected and examined under a high-power stereomicroscope (Leica M205 FA, Macquarie Park, Australia). The absence of Symbiodiniaceae autofluorescence confirmed the aposymbiotic state of theses anemones.

**1.2 Symbiodiniaceae cell density**

Cell density was measured in a CytoFLEX LX flow cytometer using the autofluorescence gating predetermined by the each Symbiodiniaceae strain. To isolate the Symbiodiniaceae, each anemone was homogenized in 500 µL of 1x phosphate-buffered saline (PBS) with 100 mg of 425- 600 µm glass beads in a tissue lyser. Sample preparation was conducted according to a method adapted from [1] and the abcam Propidium Iodide Flow Cytometry Kit protocol. To obtain freshly isolated algal symbionts, each anemone was homogenized in 500 µL of 1x phosphate-buffered saline (PBS) with 100 mg of 425- 600 µm glass beads (G8772-100G, Sigma-Aldrich) for 60 s at 30 Hz in a tissue lyser (Tissue-Lyser II, Qiagen, Hilden, Germany). The homogenates were centrifuged for 10 min at 5000 x *g* at 4 ℃ to separate the algal cells from anemone tissue, and the supernatant was collected and stored at -20 ℃ for host protein analysis.

Due to the small size of the anemones, negative protein values were found in some of the early time point samples. The method is hence not suitable for small sized anemones and host protein content is not reported in this study. The Symbiodiniaceae pellets were resuspended in 1 mL 1x PBS and centrifuged as above, then resuspended in 400 µL ice-cold 1x PBS and fixed in 800 µL ice-cold absolute ethanol. At week 9, 100 µL of the resuspension was snap frozen for ITS2 metabarcoding and the cell density of that time point was scaled to account for the 100 µL loss. Samples were left to incubate overnight at 4 ℃. To separate the cells from the ethanol, samples were first centrifuged for 10 min at 9000 x *g* at 4 ℃. The ethanol was then removed by pipetting and the samples were washed in 500 µL 1x PBS and centrifuged for 5 min at 9000 x *g* at 4 ℃. The algae were resuspended in 200 µL of 1x propidium iodide (PI) in 1x PBS with 0.1 % Triton-X114 (X114, Sigma Aldrich) and 1x RNase A. The cells were left to stain in the dark overnight at 4 ℃. One mL of Symbiodiniaceae culture of each strain was also processed in the aforementioned methods to set the autofluorescence gating for measurement.

Symbiodiniaceae density and DNA content per sample were measured using a CytoFLEX LX flow cytometer equipped with CytExpert Software (Beckman Coulter, Indianapolis, Indiana, United States). Samples were filtered through a 40 µm mesh and vortexed before being processed at a flow of 20 µL min^-1^. Gains were set manually and the calibration settings remained constant throughout the experiment. An acquisition limit of 10 000 events or a maximum acquisition volume of 100 µL (in cases of low cell density) was set for each sample. Cultures of each strain were used to define their specific autofluorescence gating window. Autofluorescence events excited by the 405 nm and 355 nm lasers at emissions 610 ± 10 nm and 405 ± 15 nm respectively, were used to gate and separate Symbiodiniaceae cells from debris left after sample preparation. Forward scatter area (FSC-A) and side scatter area (SSC-A) were used to single out the solitary cells from aggregates and remaining debris. These were then further gated on a FSC-Width and FSC-A plot to isolate the single cells. Concentration values (events µL^-1^) recorded in the gating window for single cells obtained from the CytExpert Software were used to calculate the Symbiodiniaceae density in an anemone. DNA content was measured with the intention to examine mitotic index of the algal cells to assess their growth rate. However, the number of events in a sample was below the 10 000-threshold required to obtain reliable mitotic index, hence this trait was not reported in this study.

**1.3 DNA extraction, PCR amplification and library preparation**

Sample DNA was extracted with a salting-out method [2] with the addition of a 15 min incubation with 10 mg mL^-1^ lysozyme, and 20 s bead beating at 30 Hz with 100 mg of 425- 600 sterile acid-washed glass beads. Four extraction blanks were included. Symbiodiniaceae ITS2 primers: Sym_Var_5.8S2 [5’ GTGACCTATGAACTCAGGAGT**C**GAATTGCAGAACTCCGTGAACC 3’] [3]; Sym_Var_Rev [3’ CTGAGACTTGCACATCGCAGCCGGGTTCWCTTGTYTGACT

TCATGC 5’] [4] with Illumina adapters (underlined- specific for the Walter and Eliza Hall Institute (WEHI)) were used to amplify the partial 5.8S, entire ITS2 and partial 28S rDNA genes. PCR was performed in triplicate, each containing 7.5 µL of MyTaq HSRed MasterMix (Bioline, Australia), 1.5 µL of each primer (10 µM), 3.5 µL of nuclease-free water, and 1 µL of DNA template for a 15 µL reaction. Three no template controls were included. PCR was carried out in a thermal cycler (SimpliAmp^TM^ Thermal Cycler, Thermo Fisher Scientific, Scoresby, Australia) under the following conditions: one initial denaturation cycle at 95 ℃ for 3 min, 18 amplification cycles (denaturation at 95 ℃ for 15 s, annealing at 55 ℃ for 30 s and extension at 72 ℃ for 30 s), one extension cycle at 72 ℃ for 7 min, and a final hold temperature of 4 ℃.

Triplicate PCR products were pooled and each of the 20 µL product pool were purified using Ampure XP magnetic beads. The purified DNA was then resuspended in 40 µL of nuclease-free water. Ten µL of DNA was combined with 10 µL of 2x Taq master mix (M0270S, New England BioLabs,Notting Hill, Australia), 0.5 µL of forward indexing, and 0.5 µL of reverse indexing primers. The PCR conditions were: one initial denaturation cycle at 95 ℃ for 3 min, 24 amplification cycles (denaturation at 95 ℃ for 15 s, annealing at 60 ℃ for 30 s and extension at 72 ℃ for 30 s), one extension cycle at 72 ℃ for 7 min, and a final hold temperature of 4 ℃. Product quantity and size were visualized on 1% agarose 1 x TAE agarose gel. A total of 5 µL from each reaction was pooled and a final bead clean-up was performed on the 50 µL pooled volume. Illumina MiSeq v3 sequencing was conducted at WEHI.

**1.4 Dry weight and metabolite sample processing**

Twelve anemones per Symbiodiniaceae treatment were sampled at week 9 for targeted metabolomics analysis. Two anemones of the same Symbiodiniaceae treatment were combined into a single sample and transferred to 1.5 mL RINO bead mill tubes with three 3.2 mm stainless steel beads (BioTools, Keperra, Australia) and 200 µL of 4 ℃ MilliQ water. The anemones were homogenized with a bead mill homogenizer (Precellys Evolution, Thermo Fisher Scientific) at 4800 rpm for 30 s on dry ice. Once homogenized, 400 µL of 4 ℃ MilliQ water was added to each tube and the homogenates were transferred to new pre-weighed 1.5 mL tubes. To ensure that all homogenized tissue was collected, the beads in the first tubes were washed with another 400 µL of 4 ℃ MilliQ water and the volume was added to the homogenates in the new tubes. Algal symbionts were separated from host material by centrifugation at 1900 x *g* for 10 min at 4 ℃. The host supernatant was collected and centrifuged again at 1900 x *g* for 10 min at 4 ℃ to pellet any remaining algal cells. The cleaned host fractions were transferred to fresh pre-weighed 1.5 mL tubes, frozen and freeze-dried for 24 h. The symbiont pellets were washed twice with 1 mL 4 ℃ MilliQ water, vortexed, and centrifuged at 1900 x *g* for 10 min at 4 ℃. A total of 500 µL of 4 ℃ MilliQ water was added to the resulting pellets which were frozen then freeze-dried for 24 h. Once fully dry, the host and symbiont fractions were weighed.

**1.5 Metabolite extraction**

For the host fraction, samples were extracted with 500 µL of 50 % MeOH (at -20 ℃) containing LC-MS internal standards of l-phenylalanine and succinic acid- ^13^C_6_ (Sigma-Aldrich) at 0.5 µg mL^-1^ and subsequently sonicated for 10 min at room temperature. The sonicated samples were centrifuged at 14000 x *g* for 20 min at 4 ℃ and the supernatants were collected. For the Symbiodiniaceae fraction, bead mill tubes (1.5 mL) containing the symbiont pellets, one scoop of 0.1 mm glass beads (~ 190 mg; Merck, Bayswater, Australia) and 200 µL of 50 % MeOH (at -20 ℃) with internal standards (0.1 µg mL^-1^) were bead milled at 6800 rpm for 30 s. The content was transferred to new 1.5 mL tubes and a further 300 µL of 50 % MeOH plus 0.1 µg mL^-1^ IS (0.1% at -20 ℃) was added. The samples were sonicated for 10 min at room temperature, centrifuged (14000 x *g* for 20 min at 4 ℃) and the supernatants were removed and saved. Extraction on both the host and Symbiodiniaceae fractions was repeated with 500 µL of 80 % MeOH. Samples were sonicated for 10 min at room temperature, centrifuged (14000 x *g* for 20 min at 4 ℃) and the supernatants were combined with the first extract. Fifty µL aliquots were collected from the host and symbiont samples for pooled biological quality control (PBQC). A volume of 700 µL of 100 % MeOH was added to increase lipid capture via filtration. Lipid was removed from the extracts via Captiva (Agilent Captiva EMR-Lipid cartridges, 40 mg, 96 well plate) and each sample were then concentrated in a 96 well plate in speedvac until dry.

Samples were re-suspended in 50 µL 20% MeOH and the analysis was run using the Agilent metabolomics dynamic MRM (dMRM) method (Agilent Technologies Technical Overview 3 June 2016) on the Agilent 1290 Infinity II UHPLC with an Agilent 6470 Triple Quadrupole LC-MS system with an injection volume of 4 µL. Biological blanks were run every 10 samples. Data was analyzed via MassHunter Quantitative analysis 10.0 using the host-Symbiodiniaceae PBQC, and method was applied to the samples to identify metabolite IDs. Due to laboratory access limit imposed by COVID19 restrictions, the samples had to be run in two batches, where the first batch included B1, WT10, SS- (SS5), SS+ (SS8), and the second batch included SS- (SS3, SS9) and SS+ (SS1, SS7).

**1.6 Batch correction**

After batch correction, a batch effect was still observed between the two LCMS runs in both the host and Symbiodiniaceae fractions (Figs. S1, S2). To ensure that downstream analyses were not biased by batch effects: 1) individual heat-evolved strains were not compared to each other (as this would require comparing batch one data with that of batch two), and 2) t-tests results (which utilized data from both batches) were verified with results using batch one data only. The results of using data from both batches or batch one only were similar (see Results), and all key interpretations in the Discussion remain valid based on results from either dataset.

**1.7 Sugar-related metabolites**

The 207 central carbon metabolites in this study comprise 20 sugar-related metabolites and one sugar-related pathway. These include disaccharides with two molecules of glucose (cellobiose, trehalose and d-maltose) or with one molecule of glucose and galactose (melibiose); monosaccharides (i.e., single molecular sugar: alpha-d-glucose-1-phosphate, d-sylose, l-sorbose, l-arabinose, d-mannose), where xylose has been identified as a metabolite translocated to *Exaiptasia diaphana* by Symbiodiniaceae [5]. In addition, amino sugar (galactosamine), sugar acid (galactonic acid and galactonic acid), sugar alcohol (myo-Inositol), intermediate metabolites in the amino sugar and nucleotide sugar metabolism pathway (n-acetyl-d-glucosamine 6-phosphate, d-glucose 1-phosphate, d-fructose 6-phosphate, UDP-glucose, d-glucosamine 6-phosphate and UDP-alpha-d-galactose) and another intermediate sugar metabolite (uridine 5'-diphosphogalactose) are also included.

**References**

1. Gabay Y. The cellular and physiological basis of host-symbiont specificity in a model cnidarian-dinoflagellate symbiosis. 2018. Victoria University of Wellington.

2. Wilson K, Li Y, Whan V, Lehnert S, Byrne K, Moore S, et al. Genetic mapping of the black tiger shrimp *Penaeus monodon* with amplified fragment length polymorphism. *Aquaculture* 2002; **204**: 297–309.

3. Hume BCC, D’Angelo C, Smith EG, Stevens JR, Burt J, Wiedenmann J. Symbiodinium thermophilum sp. nov., a thermotolerant symbiotic alga prevalent in corals of the world’s hottest sea, the Persian/Arabian Gulf. *Sci Rep* 2015; **5**: 8562.

4. Hume B, D’Angelo C, Burt J, Baker AC, Riegl B, Wiedenmann J. Corals from the Persian/Arabian Gulf as models for thermotolerant reef-builders: Prevalence of clade C3 Symbiodinium, host fluorescence and ex situ temperature tolerance. *Marine Pollution Bulletin* 2013; **72**: 313–322.

5. Matthews JL, Oakley CA, Lutz A, Hillyer KE, Roessner U, Grossman AR, et al. Partner switching and metabolic flux in a model cnidarian–dinoflagellate symbiosis. *Proceedings of the Royal Society B: Biological Sciences* 2018; **285**: 20182336.


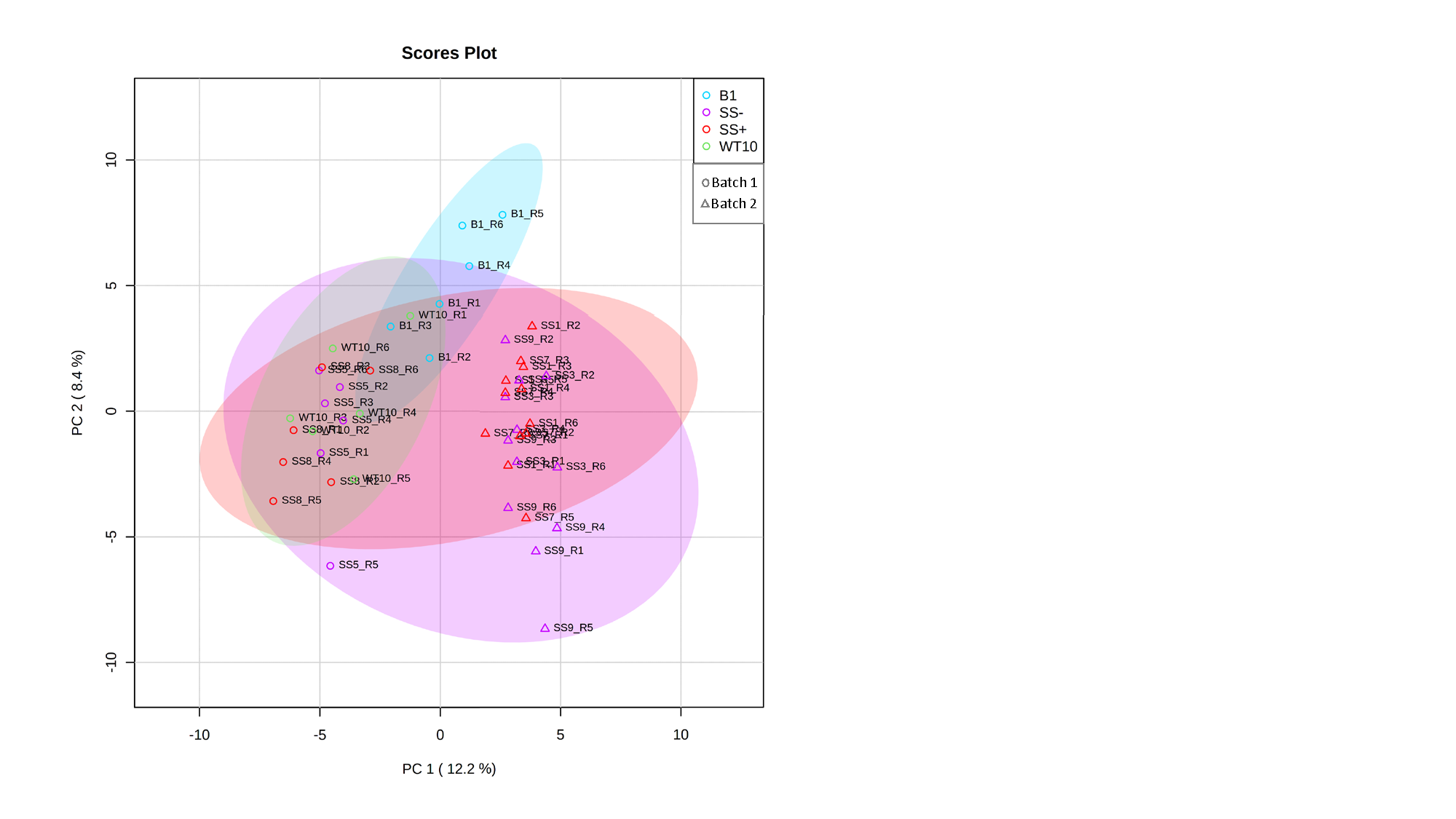


Fig. S1. PCA of all 207 central carbon metabolites from the host fraction (normalized to host dry weight) after batch correction with individual samples names. Batch effect was still observed, where batch one included SS5 (SS-), SS8 (SS+), WT10 and B1, and batch two included SS1 and SS7 (SS+), as well as SS3 and SS9 (SS-). “R” in the sample name refers the replicate number.


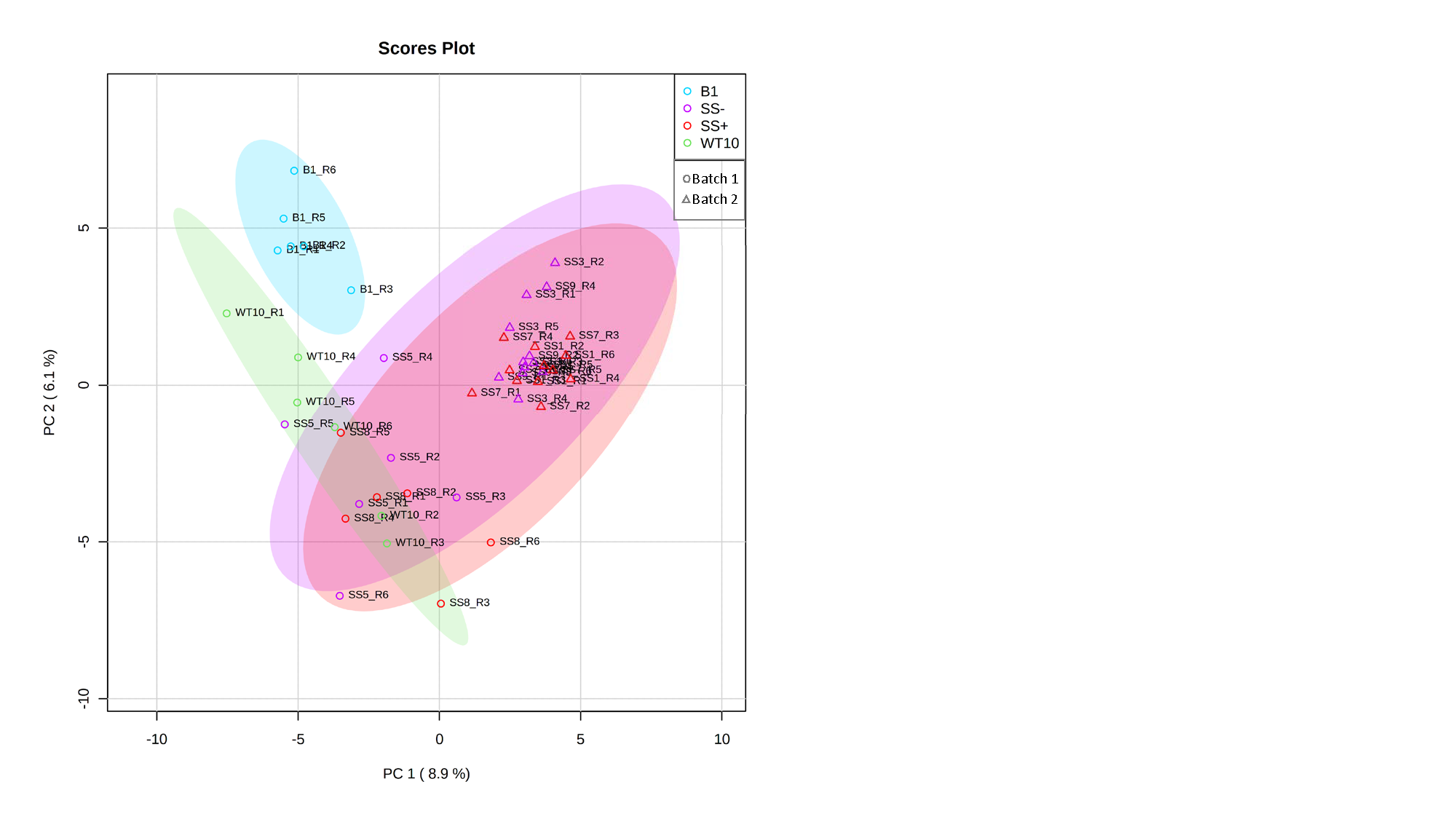


Fig. S2. PCA of all 207 central carbon metabolites from Symbiodiniaceae fraction (normalized to Symbiodiniaceae dry weight) after batch correction with individual samples names. Batch effect was still observed, where batch one included SS5 (SS-), SS8 (SS+), WT10 and B1, and batch two included SS1 and SS7 (SS+), as well as SS3 and SS9 (SS-). “R” in the sample name refers the replicate number.

Table S1. Pairwise comparison results of Symbiodiniaceae cell density per anemone at week 9. P-values were corrected using the Benjamini-Hochberg method. B1 = *B. minutum,* WT10 = wild-type *Cladocopium* C1^acro^, SS- = non-conferring heat-evolved *Cladocopium* C1^acro^, SS+ = conferring heat-evolved *Cladocopium* C1^acro^.

| **Comparison** | | | **Estimate** | **95% confidence interval** | **p_adj_** |
| --- | --- | --- | --- | --- | --- |
| WT10 | vs | B1 | -1.37 | (-2.91, 0.18) | 0.099 |
| SS+ | vs | B1* | -2.15 | (-3.42, -0.89) | <0.001 |
| SS- | vs | B1* | -1.68 | (-2.94, -0.41) | 0.006 |
| SS+ | vs | WT10 | -0.79 | (-2.05, 0.48) | 0.342 |
| SS- | vs | WT10 | -0.31 | (-1.58, 0.95) | 0.907 |
| SS- | vs | SS+ | -0.48 | (-0.42, 1.37) | 0.476 |

* indicates significant pairs.


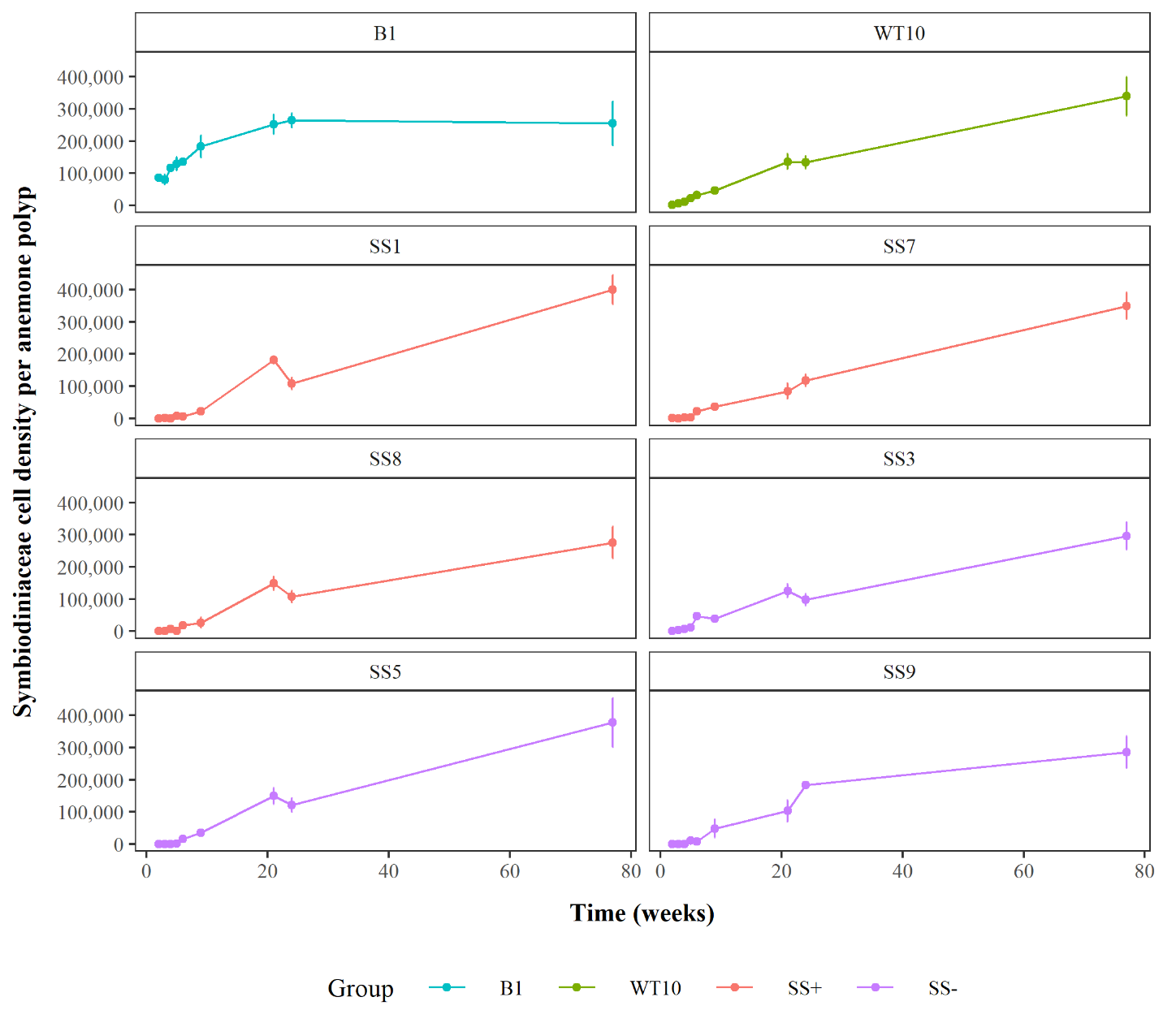


Fig. S3. Symbiodiniaceae cell density per anemone of individual Symbiodiniaceae treatment over the 77 weeks following initial inoculation. Error bars represent one standard error. B1 = *B. minutum,* WT10 = wild-type *Cladocopium* C1^acro^, SS- = non-conferring heat-evolved *Cladocopium* C1^acro^, SS+ = conferring heat-evolved *Cladocopium* C1^acro^.


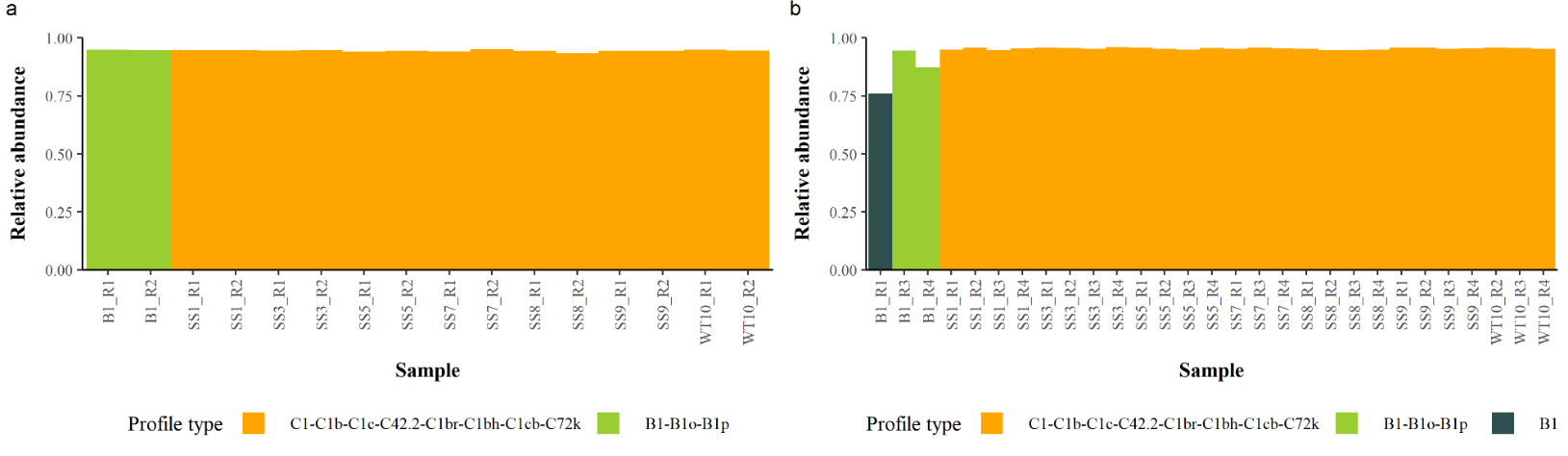


Fig. S4. Relative abundance of ITS2 profile types of the a) inocula and b) inoculated anemones at week 9. Note that the relative abundances can be less than 1 (i.e., 100%) as some sequences have no ITS2 profile.

**Supplementary Results**

**1.1 Symbiodiniaceae identity**

A total of 3.05 M reads were yielded among the 16 algal inocula samples, 32 inoculated anemones sampled at week 9, and 6 non-inoculated aposymbiotic anemones sampled at week 22. Three anemone samples that failed QC in SymPortal (i.e., one *B. minutum* (B1), one wild-type *Cladocopium* C1^acro^ (WT10), and one heat-evolved *Cladocopium* C1^acro^ strain (SS7)) were excluded from the analyses. No ITS2 profile type was identified in the non-inoculated aposymbiotic anemones, confirming that the homologous algal symbionts were completely removed from menthol bleaching and that they did not re-establish during the experiment. ITS2 profile types were also not found in extraction and PCR blanks. All *Cladocopium* C1^acro^ anemones (WT10, SS+, SS-) carried the *Cladocopium* profile type C1-C1b-C1c-C42.2-C1br-C1bh-C1cb-C72k as per their inocula. All *B. minutum* anemones had the *Breviolum* profile types B1-B1o-B1p or B1 as per their inoculum. The results confirmed that the algal symbionts present in the anemones corresponded to the algal culture used for inoculation. Note that no genetic markers are currently available to distinguish between the different *Cladocopium* C1^acro^ strains.

Table S2. The number of surviving anemones with different Symbiodiniaceae treatments of the 12 anemones set aside for survival assessment.

|  | ***B. minutum*** | **Wild-type**  ***Cladocopium* C1^acro^** | **Heat-evolved**  ***Cladocopium* C1^acro^ SS+** | | | **Heat-evolved**  ***Cladocopium* C1^acro^ SS-** | | |
| --- | --- | --- | --- | --- | --- | --- | --- | --- |
| **Week** | **B1** | **WT10** | **SS1** | **SS7** | **SS8** | **SS3** | **SS5** | **SS9** |
| Week 0 | 12 | 12 | 12 | 12 | 12 | 12 | 12 | 12 |
| Week 1 | 12 | 12 | 12 | 12 | 12 | 12 | 12 | 12 |
| Week 2 | 12 | 12 | 12 | 12 | 12 | 12 | 12 | 12 |
| Week 3 | 12 | 12 | 12 | 12 | 12 | 12 | 12 | 12 |
| Week 4 | 12 | 12 | 12 | 12 | 12 | 12 | 12 | 12 |
| Week 5 | 12 | 12 | 12 | 12 | 12 | 12 | 12 | 12 |
| Week 9 | 12 | 12 | 12 | 12 | 12 | 12 | 12 | 12 |


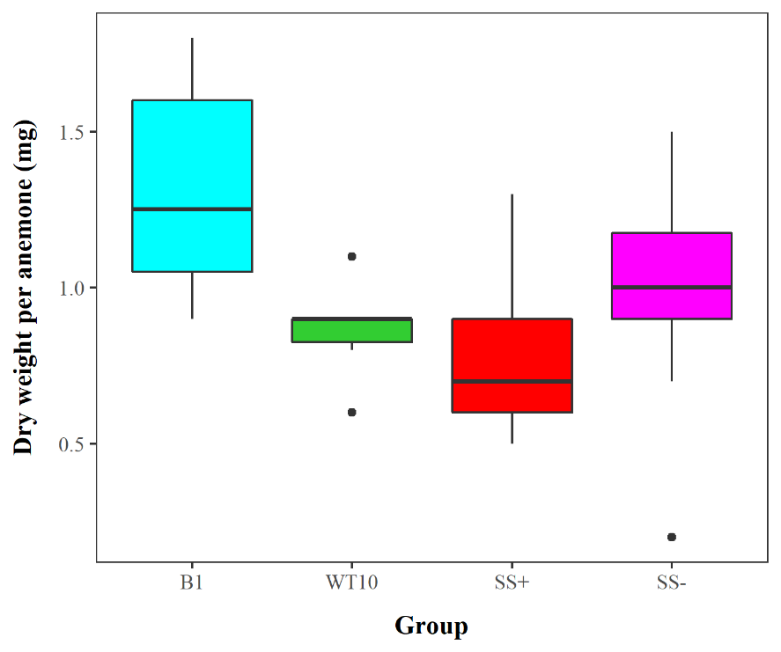


Fig. S5. Dry weight of the anemone host at week 9. B1 = *B. minutum* (n = 12)*,* WT10 = wild-type *Cladocopium* C1^acro^ (n = 12), SS- = non-conferring heat-evolved *Cladocopium* C1^acro^ (SS3, SS5, SS9, n = 12 per strain), SS+ = conferring heat-evolved *Cladocopium* C1^acro^ (SS1, SS7, SS8, n = 12 per strain).

Table S3. Pairwise comparison results on the anemone host dry weight at week 9. P-values were corrected using the Benjamini-Hochberg method. B1 = *B. minutum,* WT10 = wild-type *Cladocopium* C1^acro^, SS- = non-conferring heat-evolved *Cladocopium* C1^acro^, SS+ = conferring heat-evolved *Cladocopium* C1^acro^.

| **Comparison** | | | **diff** | **lower** | **upper** | **p_adj_** |
| --- | --- | --- | --- | --- | --- | --- |
| WT10 | vs | B1* | -0.45 | -0.86 | -0.04 | 0.025 |
| SS+ | vs | B1* | -0.56 | -0.89 | -0.22 | <0.001 |
| SS- | vs | B1* | -0.33 | -0.66 | 0.00 | 0.048 |
| SS+ | vs | WT10 | -0.11 | -0.44 | 0.23 | 0.830 |
| SS- | vs | WT10 | 0.12 | -0.21 | 0.45 | 0.784 |
| SS- | vs | SS+ | 0.22 | -0.01 | 0.46 | 0.069 |

* indicates significant pairs.


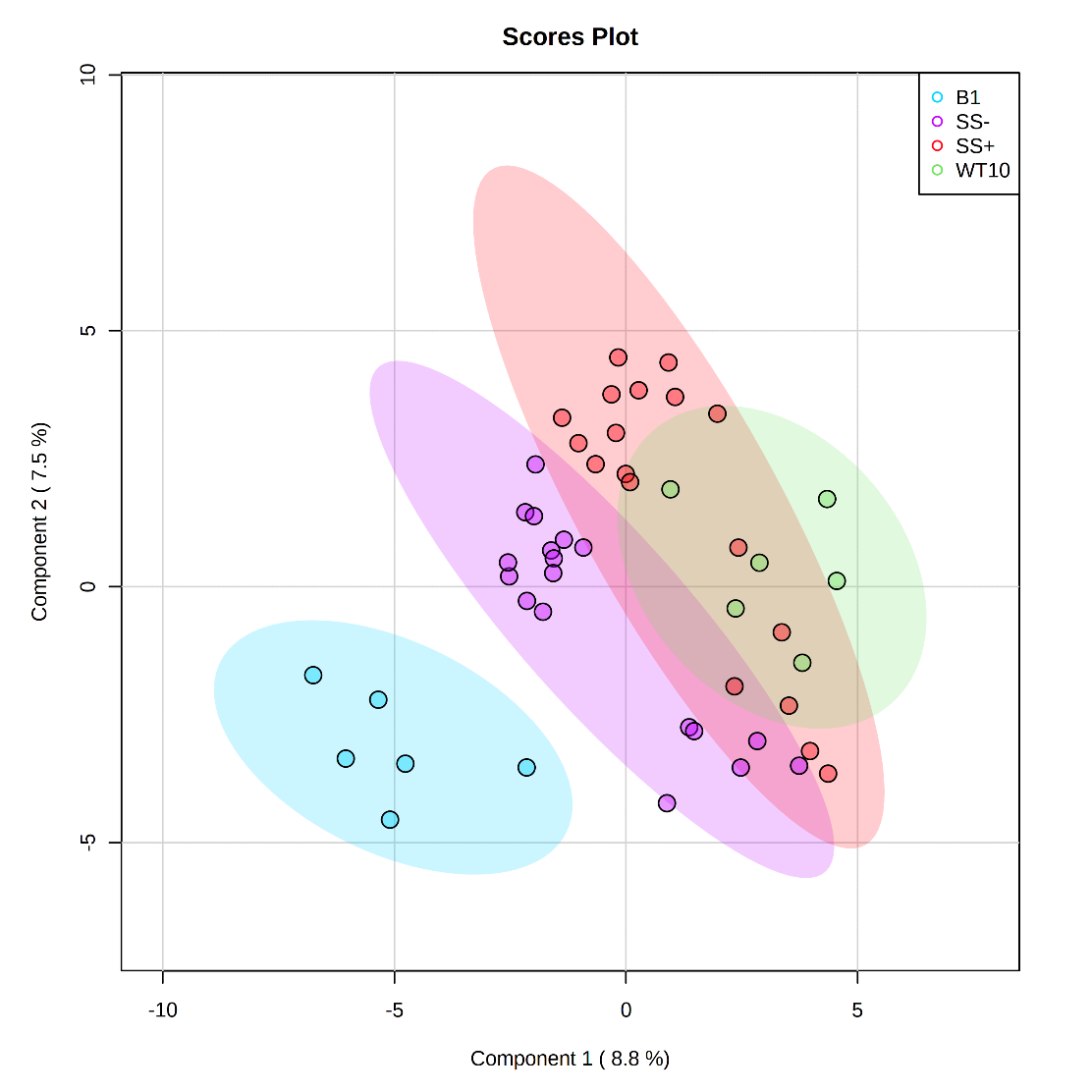


Fig. S6. PLS-DA of all 207 central carbon metabolites from the host fraction (normalized to host dry weight). Note that the PLS-DA did not pass quality control (permutation p = 0.09, Q2 < 0.2; indicating low predictive ability) and is hence not used for interpretation.

Table S4. The number of significantly different metabolites between pairs at week 9 based on t-tests. B1 = *B. minutum*, C1^acro^ = all *Cladocopium* C1^acro^ (WT10, SS+, SS-), WT10 = wild-type *Cladocopium* C1^acro^, SS- = non-conferring heat-evolved *Cladocopium* C1^acro^, SS+ = conferring heat-evolved *Cladocopium* C1^acro^, SS = all heat-evolved *Cladocopium* C1^acro^ (SS- and SS+).

| **Dataset** | **Comparison** | | | **No. of significant metabolites** |
| --- | --- | --- | --- | --- |
| Host | B1 | vs | C1^acro^ | 23 |
| Host | WT10 | vs | SS | 0 |
| Host | SS- | vs | SS+ | 0 |
| Algal symbiont | B1 | vs | C1^acro^ | 27 |
| Algal symbiont | WT10 | vs | SS | 3 |
| Algal symbiont | SS- | vs | SS+ | 0 |


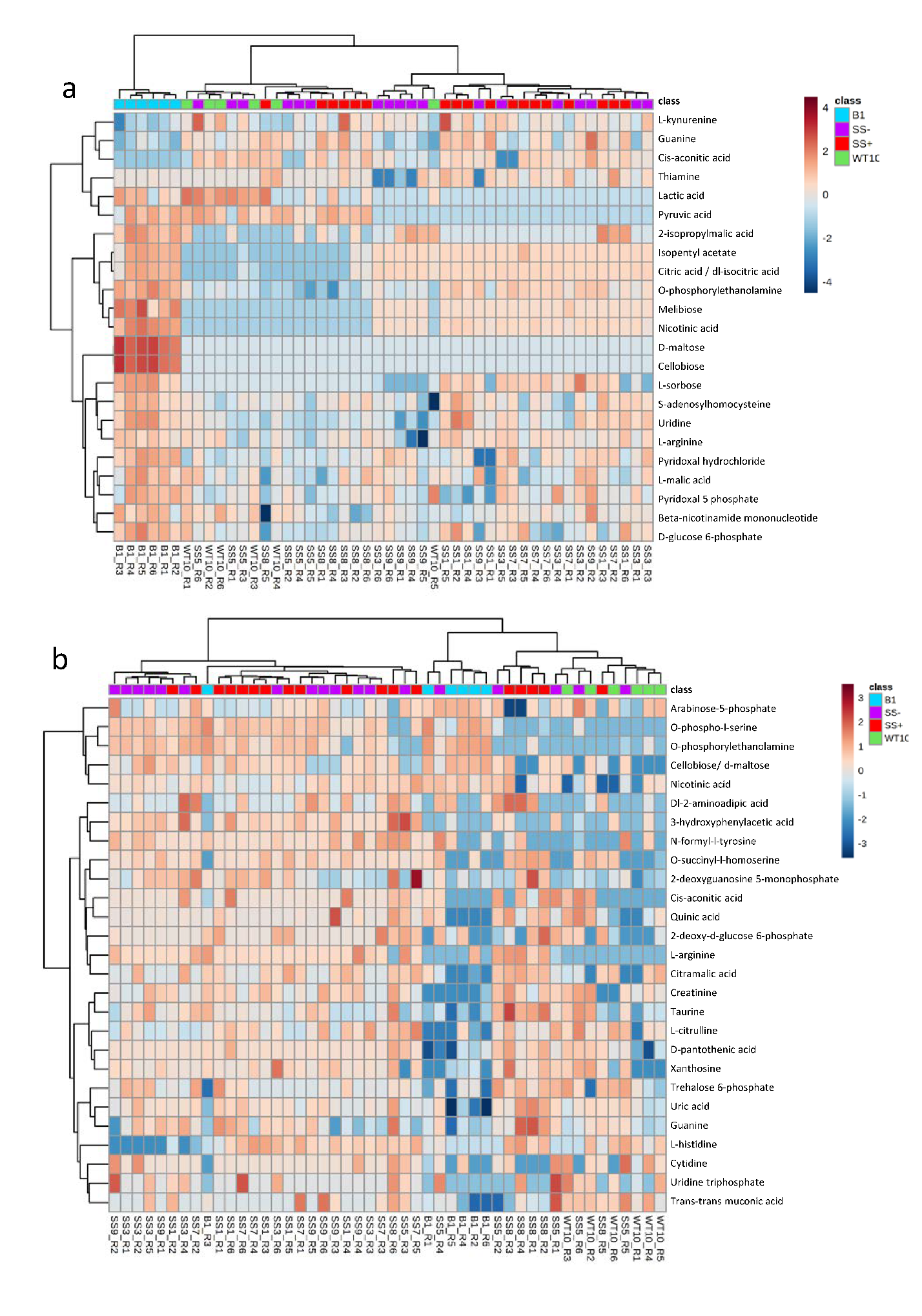


Fig. S7. Heatmap of the significantly different metabolites between the *B. minutum* and *Cladocopium* C1^acro^ groups (WT10, SS-, SS+) at week 9. a) the host fraction (normalized to host dry weight) and b) the Symbiodiniaceae fraction (normalized to Symbiodiniaceae dry weight). B1 = *B. minutum*, WT10 = wild-type *Cladocopium* C1^acro^, SS- = non-conferring heat-evolved *Cladocopium* C1^acro^, SS+ = conferring heat-evolved *Cladocopium* C1^acro^. The color scale indicates log2 fold change relative to the mean.

Table S5. Results of pathway analysis based on the 23 significantly different metabolites between the *B. minutum* and *Cladocopium* C1^acro^-anemones at week 9 in the host fraction (normalized to the host dry weight). Note that not all significant metabolites matched to a pathway.

| **Metabolite** | **Pathway Name** | **Match Status** | **p_adj_** |
| --- | --- | --- | --- |
| O-phosphorylethanolamine | Glycerophospholipid metabolism | 1/23 | <0.001 |
| Cis-aconitate acid | Glyoxylate and dicarboxylate metabolism | 1/12 | <0.001 |
| Cis-aconitate acid | Citrate cycle (TCA cycle) | 1/20 | <0.001 |
| Myo-inositol | Inositol phosphate metabolism | 1/9 | 0.001 |
| Myo-inositol | Phosphatidylinositol signaling system | 1/12 | 0.001 |
| Uridine | Pyrimidine metabolism | 1/28 | 0.001 |
| Nicotinic acid | Purine metabolism | 1/45 | 0.003 |

Table S6. T-test results and fold changes of significant metabolites between the *B. minutum*- and *Cladocopium* C1^acro^-anemones (WT10, SS-, SS+) at week 9 from the host fraction of batch one only (normalized to host dry weight). Bold indicates metabolites that match with significant metabolites when both batch one and batch two data were used (Table 3).

| **Metabolites** | **t.stat** | **p_adj_** | **FC** | **log2(FC)** |
| --- | --- | --- | --- | --- |
| **D-maltose** | 31.5 | <0.001 | 7.45 | 2.90 |
| **Nicotinic acid** | 31.4 | <0.001 | 39.71 | 5.31 |
| **Cellobiose** | 30.5 | <0.001 | 7.46 | 2.90 |
| **Melibiose** | 15.9 | <0.001 | 28.94 | 4.85 |
| **L-sorbose** | 13.6 | <0.001 | 14.01 | 3.81 |
| **Myo-inositol** | 9.5 | <0.001 | 11.66 | 3.54 |
| **Isopentyl acetate** | 8.2 | <0.001 | 76.85 | 6.26 |
| **O-phosphorylethanolamine** | 8.1 | <0.001 | 20.82 | 4.38 |
| **Pyridoxal hydrochloride** | 7.9 | <0.001 | 4.08 | 2.03 |
| **Citric acid / dl-isocitric acid** | 7.3 | <0.001 | 68.87 | 6.11 |
| **Uridine** | 6.8 | <0.001 | 2.41 | 1.27 |
| **4-pyridoxic acid** | 6.6 | <0.001 | 2.89 | 1.53 |
| **Salicylic acid** | 5.6 | <0.001 | 3.33 | 1.73 |
| **Thymidine** | 5.4 | <0.001 | 2.39 | 1.25 |
| **L-arabinose** | 5.2 | <0.001 | 18.03 | 4.17 |
| **2-isopropylmalic acid** | 4.8 | 0.001 | 9.83 | 3.30 |
| **D-glucose 6-phosphate** | 4.8 | 0.001 | 2.19 | 1.13 |
| Taurocholic acid | 4.5 | 0.002 | 398.60 | 8.64 |
| **Cis-aconitic acid** | -4.3 | 0.003 | 0.08 | -3.65 |
| L-homocystine | 3.9 | 0.008 | 7.81 | 2.97 |
| **Guanine** | -3.8 | 0.009 | 0.47 | -1.10 |
| L-threonine | 3.8 | 0.010 | 5.65 | 2.50 |
| Trans-aconitic acid | -3.4 | 0.021 | 0.01 | -7.21 |
| 2-2-dimethyl succinic acid / adipic acid | 3.3 | 0.027 | 1.83 | 0.87 |
| Glyceric acid | 3.3 | 0.028 | 6.15 | 2.62 |
| Uridine diphosphate glucose | 3.2 | 0.034 | 4.35 | 2.12 |
| Deoxyadenosine monophosphate | 3.1 | 0.035 | 5.30 | 2.41 |
| Inosine | 3.1 | 0.035 | 2.40 | 1.26 |
| 2-phosphoglyceric acid | 3.0 | 0.044 | 1.65 | 0.73 |
| L-phenylalanine | 2.9 | 0.049 | 1.47 | 0.55 |
| Uric acid | -2.9 | 0.049 | 0.56 | -0.83 |


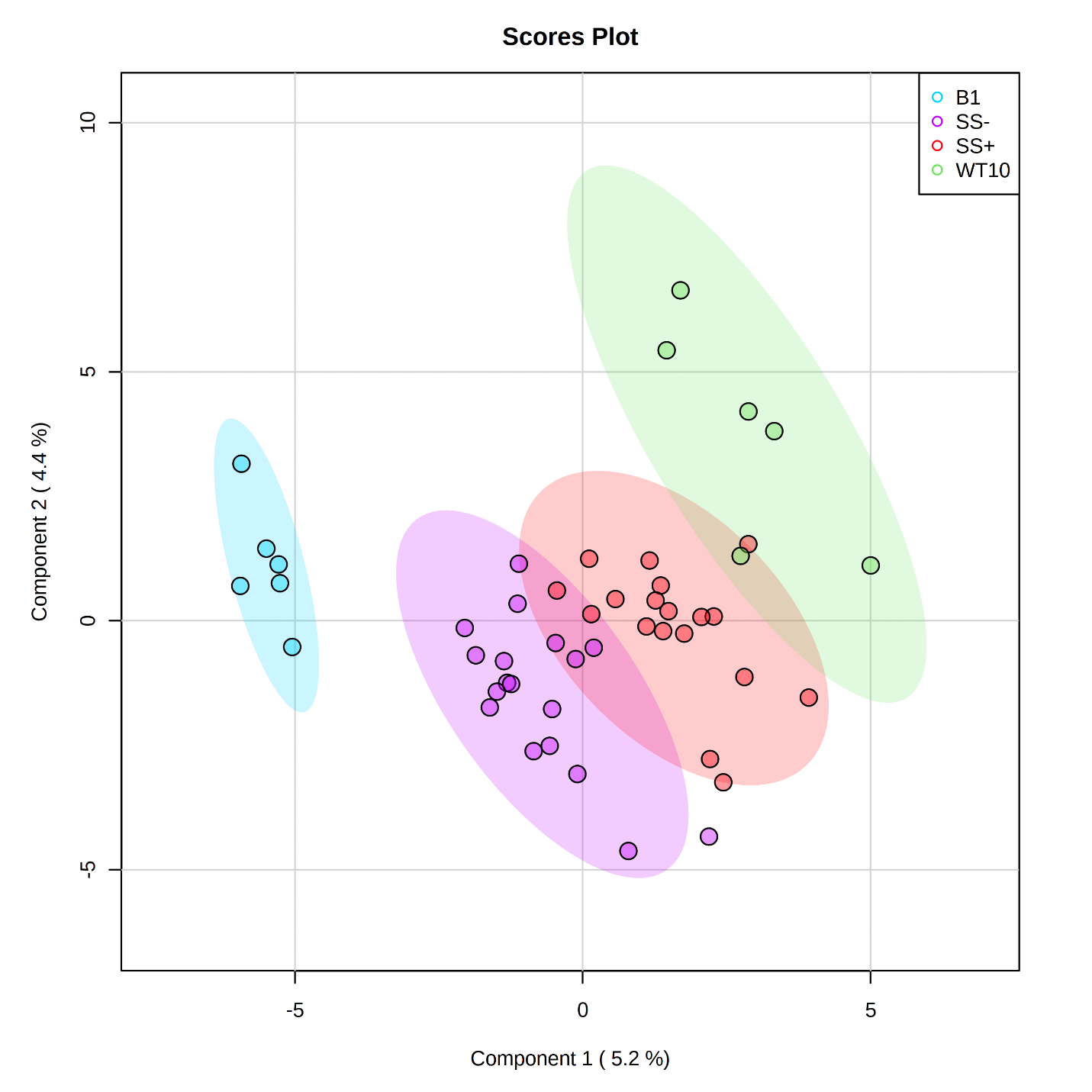


Fig. S8. PLS-DA of all 207 central carbon metabolites from the Symbiodiniaceae fraction (normalized to Symbiodiniaceae weight). Note that the PLS-DA did not pass quality control (permutation p = 0.45, Q2 ~ 0.3; indicating low predictive ability) and is therefore not discussed further.

Table S7. Results of pathway analysis based on the 27 significantly different metabolites between the *B. minutum* and *Cladocopium* C1^acro^ groups at week 9 in the Symbiodiniaceae data. Note that not all significant metabolites matched to a pathway.

| **Metabolite** | **Pathway** | **Match Status** | **p_adj_** |
| --- | --- | --- | --- |
| Uridine triphosphate, cytidine triphosphate | Pyrimidine metabolism | 2/28 | <0.001 |
| Cis-aconitate acid,  L-glutamic acid | Glyoxylate and dicarboxylate metabolism | 2/12 | 0.004 |
| Guanine, xanthine | Purine metabolism | 2/45 | 0.005 |
| D-pantothenic acid | Pantothenate and CoA biosynthesis | 1/11 | 0.004 |
| O-phosphorylethanolamine | Glycerophospholipid metabolism | 1/23 | 0.004 |
| Cis-aconitate | Citrate cycle (TCA cycle) | 1/20 | 0.005 |
| Isopentenyl pyrophosphate | Terpenoid backbone biosynthesis | 1/16 | 0.005 |
| L-glutamic acid | Arginine biosynthesis | 1/7 | 0.005 |
| L-glutamic acid | Alanine, aspartate and glutamate metabolism | 1/12 | 0.005 |
| L-glutamic acid | Glutathione metabolism | 1/21 | 0.005 |
| L-glutamic acid | Porphyrin and chlorophyll metabolism | 1/19 | 0.005 |
| L-glutamic acid | Nitrogen metabolism | 1/3 | 0.005 |
| L-glutamic acid | Aminoacyl-tRNA biosynthesis | 1/46 | 0.005 |


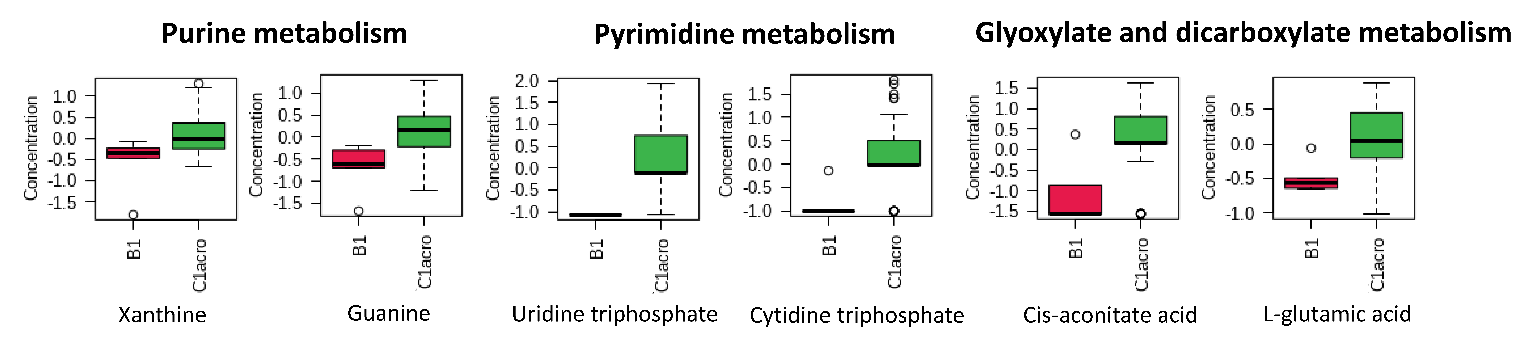


Fig. S9. Normalized and mean centered concentrations (i.e., concentration of two anemones per mg of Symbiodiniaceae dry weight) of the metabolites in pathways with two matches in the Symbiodiniaceae fraction. B1 = *B. minutum*, C1^acro^ = *Cladocopium* C1^acro^ groups (WT10, SS+, SS-).

Table S8. T-test results and fold changes of the three metabolites significantly different between the wild-type *Cladocopium* C1^acro^ (WT10) and heat-evolved *Cladocopium* C1^acro^ (SS- and SS+) at week 9 from the Symbiodiniaceae fraction (normalized to Symbiodiniaceae dry weight).

| **Metabolite** | **t.stat** | **p_adj_** | **FC** | **log2(FC)** |
| --- | --- | --- | --- | --- |
| L-arginine | 5.11 | 0.002 | 307.36 | 8.26 |
| O-phospho-l-serine | 4.05 | 0.024 | 369.72 | 8.53 |
| N-formyl-l-tyrosine | 3.77 | 0.037 | 12.46 | 3.64 |

Table S9. T-test results and fold changes of significant metabolites between the *B. minutum* and *Cladocopium* C1^acro^ (WT10, SS-, SS+) at week 9 from the Symbiodiniaceae fraction of batch one only (normalized to Symbiodiniaceae dry weight). Bold indicates metabolites that match with significant metabolites when both batch one and batch two data were used (Table 4).

| **Metabolites** | **t.stat** | **p_adj_** | **FC** | **log2(FC)** |
| --- | --- | --- | --- | --- |
| **Alpha-d-glucose-1-phosphate** | 7.12 | <0.001 | 2.49 | 1.32 |
| **Melibiose** | 7.19 | <0.001 | 11.6 | 3.54 |
| **O-phospho-l-serine** | 7.87 | <0.001 | 295.83 | 8.21 |
| **O-phosphorylethanolamine** | 9.71 | <0.001 | 192.49 | 7.59 |
| **Uric acid** | -5.82 | <0.001 | 0.07 | -3.76 |
| **L-arabitol / xylitol** | -4.62 | 0.005 | 0.16 | -2.68 |
| **Myo-inositol** | 3.92 | 0.019 | 2.65 | 1.41 |
| **Trehalose 6-phosphate** | -3.91 | 0.019 | 0.10 | -3.32 |
| **L-glutamic acid** | -3.75 | 0.025 | 0.43 | -1.22 |
| **D-mannose/ l-sorbose** | 3.52 | 0.036 | 2.19 | 1.13 |
| **Guanine** | -3.52 | 0.036 | 0.23 | -2.10 |
